# Thermally Triggered Double Emulsion‐Integrated Hydrogel Microparticles for Multiplexed Molecular Diagnostics

**DOI:** 10.1002/advs.202408158

**Published:** 2025-01-16

**Authors:** Eui Ju Jeon, Seungwon Jung, Yoon‐ha Jang, Seoyoung Lee, Song‐Ee Choi, So Young Jeon, Lankyeong Yoon, Bong Kyun Kim, Tae Jong Kim, Kuenyoul Park, Seok Chung, Yong Shin, Sung‐Han Kim, Heungsup Sung, Sang Kyung Kim

**Affiliations:** ^1^ Center for Advanced Biomolecular Recognition Biomedical Research Division Korea Institute of Science and Technology (KIST) Seoul 02792 Republic of Korea; ^2^ Department of Mechanical Engineering Korea University Seoul 02841 Republic of Korea; ^3^ Department of HY‐KIST Bio‐convergence Hanyang University Seoul 04763 South Korea; ^4^ Department of Laboratory Medicine Sanggye Paik Hospital School of Medicine Inje University Seoul 01757 Republic of Korea; ^5^ Department of Biotechnology College of Life Science and Biotechnology Yonsei University Seoul 03722 Republic of Korea; ^6^ Department of Infectious Diseases Asan Medical Center University of Ulsan College of Medicine Seoul 05505 Republic of Korea; ^7^ Department of Laboratory Medicine Asan Medical Center University of Ulsan College of Medicine Seoul 05505 Republic of Korea; ^8^ KHU‐KIST Department of Converging Science and Technology Kyung Hee University Seoul 02447 Republic of Korea

**Keywords:** double emulsion, hydrogel, multiplexed assay, thermo‐responsive release

## Abstract

During the COVID‐19 pandemic, reverse transcription‐quantitative polymerase chain reaction (RT‐qPCR) has been recognized as the most reliable diagnostic tool. However, there is a need to develop multiplexed assays capable of analyzing multiple genes simultaneously to expand its application. To address this, a multiplexed RT‐qPCR using a double emulsion (DE)‐based carrier and a polymer microparticle reactor, termed primer‐incorporated network tailored with Taqman probe (TaqPIN) is developed. The DE securely stores nucleic acid reagents like primers and probes within the polymer network until heating releases them for the reaction. The TaqPIN RT‐qPCR demonstrates an amplification efficiency of 93.8% and can detect as few as 20 copies/µL. By loading the multiple microparticles into a single reaction, a multiplexed assay with only one optical channel is enabled. In practice, a nine‐plex assay is designed to distinguish between variants of the severe acute respiratory syndrome coronavirus 2 (SARS‐CoV‐2). Even subtle variations of a single nucleotide can be simultaneously detected. Testing on 75 nasopharyngeal swab samples yields 100% sensitivity and specificity for SARS‐CoV‐2 detection and 94% accuracy in variant discrimination.

## Graphical abstract

This paper presents the development of a multiplexed RT‐qPCR using a double emulsion (DE)‐based carrier and hydrogel microparticle reactor, termed TaqPIN. The DE, enhanced by a hydrogel network, securely stores nucleic acid reagents until heating releases them for reaction. TaqPIN assay shows 93.8% amplification efficiency, detecting 20 copies/µL, enabling a nine‐plex assay to distinguish SARS‐CoV‐2 variants with high sensitivity and specificity.

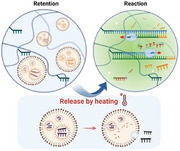

## 1 Introduction

Emulsions are fundamentally composed of two immiscible liquids, typically oil, and water, and are stabilized by emulsifying agents that prevent the coalescence of droplets, thus maintaining the integrity of the emulsion. The emulsions offer effective encapsulation, enabling the entrapment of hydrophobic or hydrophilic compounds within distinct phases.^[^ ^1^, ^2^ ^]^ The encapsulation capabilities of emulsions safely deliver sensitive materials to their destination, improving stability during storage. This unique ability of emulsions is pivotal in various applications, ranging from food products to pharmaceuticals.^[^ ^3^ ^]^ Beyond these traditional areas, emulsions have found applications in biochemical reactions over the past decades.^[^ ^4^ ^]^

In the realm of biochemical reactions, emulsions have emerged as an attractive tool, particularly in advanced molecular biology techniques.^[^ ^5^ ^]^ Droplet digital polymerase chain reaction (ddPCR) employed emulsions to create thousands of individual droplet reactors, enabling highly sensitive and precise quantification of nucleic acid amounts in samples. The integration of emulsion technology with microdroplet microfluidics has revolutionized high‐throughput analysis, crucial in fields such as drug screening, single‐cell analysis, and personalized medicine.^[^ ^6^ ^]^ This synergistic combination allows for rapid and efficient analysis of large numbers of samples, underscoring the importance of emulsions in modern biochemical analysis.^[^ ^7^ ^]^ While emulsions are extensively used to create independent reaction environments, their potential for controlled release applications in biochemical settings has not been as thoroughly explored. This contrasts with the fields of food and cosmetics, where controlled release is commonly applied. In biochemical applications, only a limited number of stimuli, such as pH changes, the addition of surfactants, or osmotic shock, have been used to trigger the release of reagents during reactions.^[^ ^8^ ^]^ However, these methods can still alter the environment in ways that may interfere with sensitive biochemical processes. Therefore, it is essential to develop specific release‐triggering mechanisms that either utilize the reaction conditions themselves or avoid disrupting the reactions within these systems.^[^ ^9^ ^]^

Here we utilize emulsions as thermo‐responsive carriers to store target‐specific PCR reagents, such as primers and probes, and release them promptly by heat. A water‐in‐oil‐in‐water double emulsion (W1/O/W2 DE) stabilized by oleophilic (W1/O) and hydrophilic (O/W2) surfactants is used as the carrier. Increasing the temperature for PCR, which has a relatively high operating temperature between 55 and 95 °C, can destabilize the interface of the emulsion, causing the contents of the W1 phase to be released to the outside. To fabricate a target‐specific reactor with DEs, the DEs are integrated into the hydrogel microparticles, which act as target‐specific independent microreactors called a primer‐incorporated network tailored with the Taqman probe (TaqPIN). DE can be used to preserve reagents during the microparticle storage and the reverse transcription (RT) process, resulting in a ready‐to‐use microreactor with full integration of target‐specific reagents. In addition, the integration of DEs in the hydrogel network dramatically enhanced its storing and releasing characteristics of DEs owing to its intertwined and complementary structure. In a microreactor of TaqPIN, RT‐PCR showed an amplification efficiency of 93.8% and a limit‐of‐detection of 20 genome equivalents (GE)/µL. The target‐specific configuration of the TaqPIN leads to multiplexed RT‐qPCR by loading multiple TaqPINs into a single PCR chip. As a practical utility, the nine‐plex assay was configured for the detection of severe acute respiratory syndrome coronavirus 2 (SARS‐CoV‐2) and the discrimination of its variants at the same time. The assay demonstrated 100% sensitivity and specificity in detecting SARS‐CoV‐2 from 75 nasopharyngeal swab samples. The discrimination of six different SARS‐CoV‐2 variants, including Alpha, Beta, Gamma, Delta, and Omicron was clinically demonstrated with 94% concordance with the result of sequencing for variant identification. It also simultaneously distinguished between two variant strains that differed by only a single nucleotide.

## 2 Results

### 2.1 Working Principle of TaqPIN RT‐qPCR

**Figure 1:**
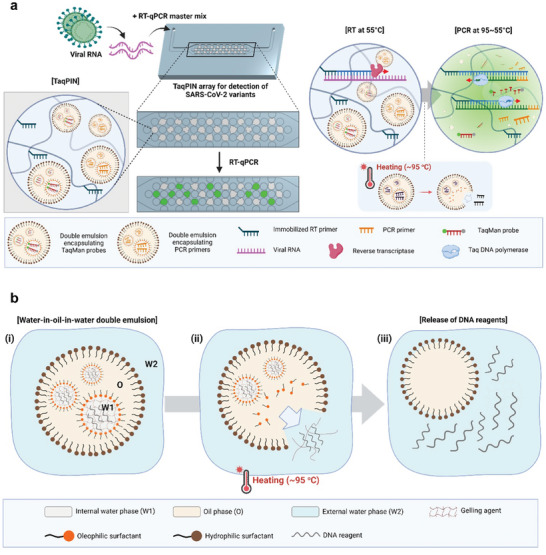
Schematic illustrating SARS‐CoV‐2 detection and viral strain discrimination using the TaqPIN assay. a) The TaqPIN assay facilitates the simultaneous detection of multiple nucleic acid targets by simply introducing a sample and RT‐qPCR mastermix. RT occurs with primers immobilized on a hydrogel network in TaqPIN at 55 °C. Subsequently, PCR is conducted with the repetition of the temperature between 55 and 95 °C, with primers and probes released from the DEs through heating. b) Schematic representation of the thermally triggered release of reagents from DE. At high temperatures, the encapsulated reagents are released due to the increased instability of the surfactant‐mediated interface. Created with BioRender.com.

In probe‐based one‐step RT‐qPCR, three different target‐specific nucleic acid reagents are required: two primers flanking the region of interest of the target nucleic acid, and a probe that produces a target‐specific fluorescence signal. In the TaqPIN assay, each microparticle contains all three target‐specific reagents to construct a target‐specific reactor (**Figure** **1**a). Of these, only the primer that also functions as the RT primer is utilized for the synthesis of the first cDNA during the RT process, while all three primers participate in the subsequent PCR process. TaqPIN employs the primer immobilized on the polymer network to efficiently reverse transcribe the target RNA while storing the other primer and probe in an inactive state within the thermo‐responsive DEs of the microparticle. Upon initiation of PCR, the elevated temperature triggers the release of the internal reagents from the DEs into the microreactor space of the TaqPIN.

Just before the start of the assay, TaqPIN microparticles containing all target‐specific reagents are soaked with a mixed solution of the target sample and master mix, which includes common reagents such as reverse transcriptase, Taq polymerase, and dNTPs. Upon introducing the target RNA into the TaqPIN and adjusting the reaction temperature, the target RNA binds to the RT primer immobilized on the polymer network, initiating the synthesis of the first cDNA (i.e., the RT process). Only the immobilized RT primers participate in the reverse transcription of target RNA, while the other primers remain stationary in DEs, thereby avoiding unwanted side reactions. Otherwise, during the RT incubation at a constant temperature for an extended period, abundant primers may incorrectly bind to some background RNA, which can negatively impact the sensitivity and specificity of the assay.

After the 8‐min RT process, the temperature rises to 95 °C to transit the phase from RT to PCR, deactivating reverse transcriptase and activating Taq polymerase. The high temperature necessary for PCR also destabilizes the thermo‐responsive DEs in TaqPIN, resulting in the release of the primers and probes stored inside. The released reagents, now free‐moving molecules, effectively participate in the PCR process, amplifying the cDNAs and generating an ideal real‐time amplification signal. If all the primers were immobilized, as in conventional solid‐phase PCR, the amplification rate would have been significantly slower, thereby losing all the superior aspects of real‐time PCR. Thus, for a successful TaqPIN PCR, DEs are the most critical elements, as they must effectively encapsulate the reagents, stably retain them until the reaction, and release them at the right time (Figure 1b).

### 2.2 Optimization of DE Composition

**Figure 2:**
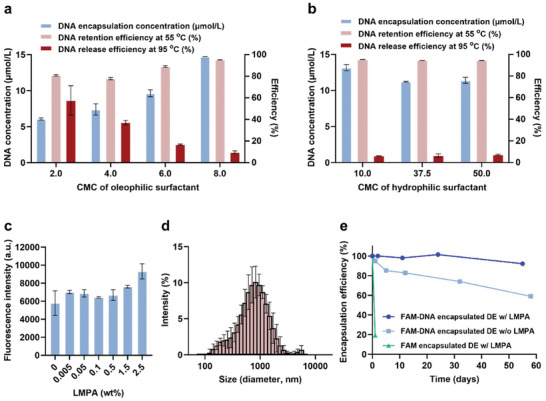
DEs as thermo‐responsive DNA carriers. a,b) DNA encapsulation concentration (left y‐axis), and retention and release efficiencies (right y‐axis) of the DEs depending on the concentration of oleophilic surfactant (a) and hydrophilic surfactant (b) (n = 3). c) Fluorescence intensity of encapsulated FAM‐DNA in DEs as a function of LMPA concentration. The error bars represent standard deviations (n = 3). d) Size distribution of DEs analyzed by DLS. e) Storage stability of DEs with (w/) and without (w/o) LMPA. Tests were conducted using DEs encapsulating FAM or FAM‐DNA. a.u., arbitrary unit.

The DEs were prepared using fluorinated oil as oil phase with the aid of oleophilic and hydrophilic surfactants as emulsifiers and stabilizers at the internal W1/O and external O/W2 interfaces, respectively (Figure 1b (i)). Specifically, 008‐fluorosurfactant was blended with HFE‐7500 oil as a surfactant for W1/O (Figure S1, Supporting Information). Low‐melting‐point agarose (LMPA) was dissolved in water (W1) as a gelling agent to render the internal aqueous droplet more viscoelastic (Figure S2, Supporting Information). W1/O emulsion was produced via tip sonication at 50 W for 45 sec, followed by brief centrifugation to separate and remove excess oil. Then, the remained W1/O emulsion was mixed with PBS buffer with Tween‐20 (W2), and another tip sonication was conducted at 50 W for 10 sec to complete the W1/O/W2 DEs. Since the surfactants used for DE formation determine the stability of the DEs, their composition for preparing the DEs was first investigated. Fluorescein (FAM)‐labeled DNA (FAM‐DNA) was used as a representative of encapsulated nucleic acid reagents to evaluate DEs as carriers. Through the measurement of the change in fluorescence intensity, the efficiencies of the encapsulation, the retention, and the release of the DEs were estimated in sequence (Tables S1,S2, Supporting Information). An insufficient quantity of either surfactant or a concentration equal to or below 1 critical micelle concentration (CMC), led to the unstable formation of the DEs (Figures S3a,S4a, Tables S1,S2, Supporting Information). In the case of an oleophilic surfactant at the internal W1/O interface, a concentration of surfactant between 2 and 8 CMC was effective in forming stable DEs (**Figure** **2**a; Figure S3b, Supporting Information). However, a concentration of oleophilic surfactant equal to or greater than 12 CMC was found to be detrimental to the separation of the emulsion from the oil, ultimately resulting in a poor yield of the DEs (Figure S3c, Supporting Information). In hydrophilic surfactant at the external O/W2 interface, a surfactant concentration of 1 CMC resulted in the merging of the DEs and the formation of large emulsions. In contrast, hydrophilic surfactant concentrations of 10–50 CMC reliably formed DEs with stable performance (Figure 2b; Figure S4b–d, Supporting Information). With the above conditions, the encapsulation concentration greater than 10 µmol/L, the retention efficiency of ≈95.5% at 55 °C (temperature for RT), and the release efficiency of ≈9.3% at 95 °C (temperature for PCR) were shown (Table S2, Supporting Information). The RT stage was set to 55 °C, as close as possible to the PCR temperature, to rigorously evaluate the performance of DEs in switching from storage to release. Notably, the release occurred immediately upon ramping to 95 °C for PCR, which is crucial for ensuring an effective reaction by supplying sufficient reagents(Figure S5, Supporting Information). This rapid release distinguishes our releasing mechanism from slower releases triggered by other stimuli that could potentially compromise reaction efficiency.^[^ ^8^ ^]^ In addition to optimizing surfactants, the use of LMPA in the internal aqueous phase enhanced the mechanical strength of the emulsion, enabling it to withstand the ultrasonication of high shear forces, and then enabled the encapsulation of more reagents. Figure 2c showed that a higher concentration of gel in the internal phase led to higher encapsulation efficiency. Since >2.5% (w/v) of gel brought about difficulty in handling due to poor solubility and high viscosity, 2.5% (w/v) of LMPA was chosen throughout this study. The dimensions of the DEs fabricated with the optimized composition of DEs were measured by dynamic light scattering (DLS) and a bright field microscope. The results demonstrated a Gaussian distribution with a peak at ≈900 nm as shown in Figure 2d. The variation of the diameter was calculated to be 6.50%, and the batch‐to‐batch variation was calculated to be 5.12%.

### 2.3 Long‐Term Storage Stability of DEs

The thermodynamic stability of W1/O/W2 DEs may be threatened even in the absence of external perturbation because of various destabilization phenomena. These include flocculation in oil droplets, coalescence between internal W1 phases or between internal W1 and external W2 phases, diffusion between W1 and W2 phases, and Ostwald ripening.^[^ ^1^, ^10^ ^]^ While DEs exhibit excellent encapsulation and thermo‐responsive release properties, as evidenced above, it is equally important to ensure the safe storage of their cargo for a certain period of time until the release is triggered by intention. Diffusion is one of the most critical causes for the migration of chemical species from the internal phase to the external phase or vice versa.^[^ ^11^ ^]^ In contrast to small molecules, such as fluorescence dyes present in the W1 phase, which readily diffused out to the W2 phase in our DE system over time, primers and probes encapsulated in DE for this study showed negligible diffusion (Figure 2e). This may be attributed either to the larger size (≈6 kDa) or to the negatively charged chemical backbone of the primers and the probes, rendering them difficult to diffuse through the oil layer. Therefore, the coalescence between the W1 and W2 phases is considered the primary cause of the loss of reagents in this study, which results in the W/O/W DE transforming into a simple O/W emulsion. The gel filling in the W1 phase which was employed to enhance mechanical strength during the production of DEs, also improved the stability of the DEs afterward, since the internal aqueous emulsions with high viscosity were less prone to coalescence.^[^ ^12^ ^]^ Figure 2e showed that the gel in the internal phase effectively resisted the escape of the reagents over storage time in comparison to DEs without gel inside, up to ≈2 months at 4 °C storage. The results indicated that the retention efficiency of DEs without internal gel was 59% at 58 days after manufacture, whereas DEs with internal gel showed an efficiency of 92% at the same time point. This evidence suggests that the internal gel played an integral role in enhancing the mechanical strength, enabling a greater capacity for encapsulating reagents and extending their storage period.

### 2.4 Formation of TaqPIN DEs

**Figure 3:**
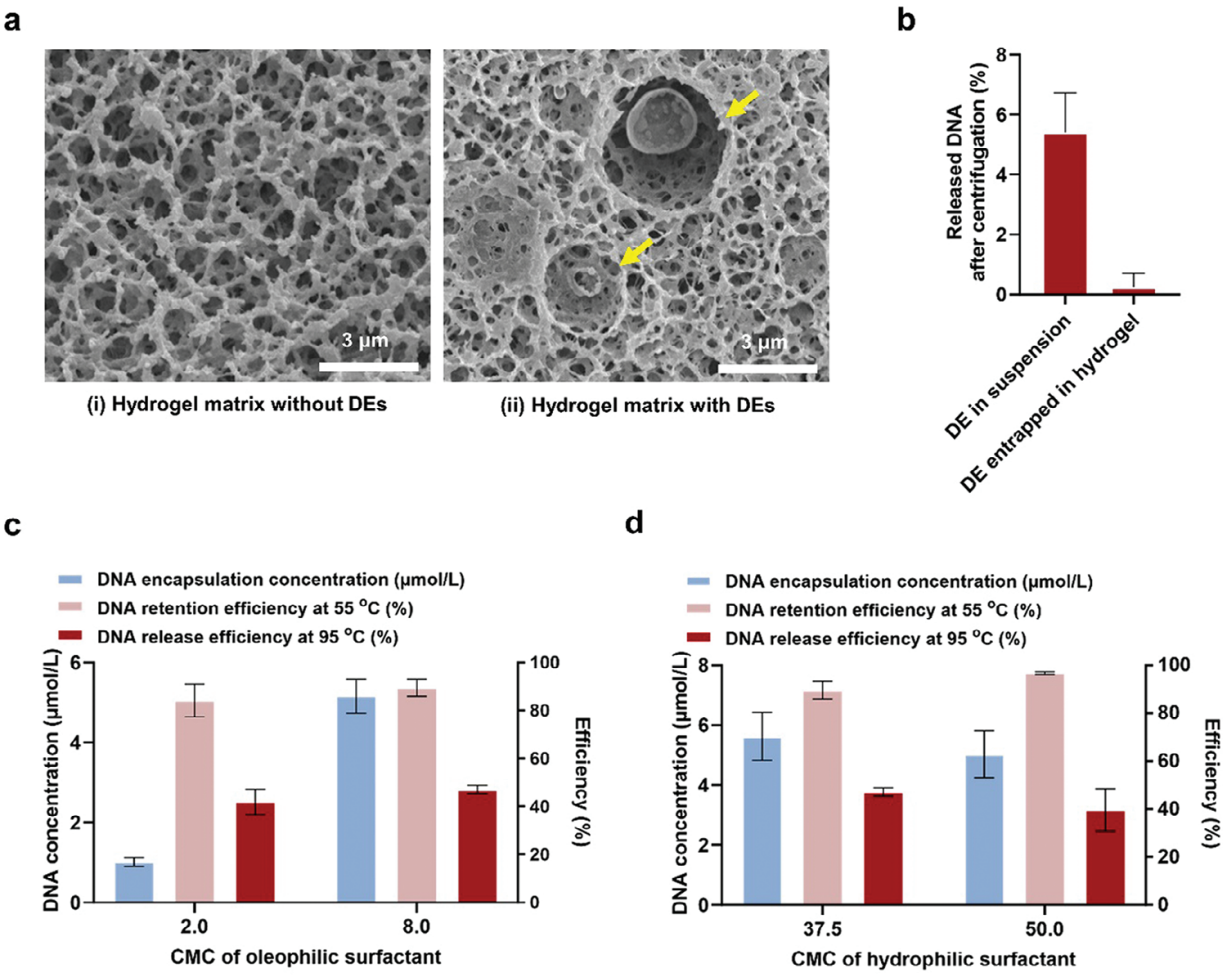
Preparation of DE‐integrated TaqPIN. a) Cryo‐SEM images of the hydrogel without DEs i) and with DEs ii). The yellow arrows indicate the space of DEs entrapped in a hydrogel matrix. DEs may be evaporated and shrunk during sample preparation for cryogenic imaging. b) Assessment of the stability of DEs in hydrogel under shear stress (centrifugation, n = 3). c,d) DNA encapsulation concentration (left y‐axis), and retention and release efficiencies (right y‐axis) of DEs trapped in hydrogel depending on the concentration of oleophilic surfactant (c) and hydrophilic surfactant (d) (n = 10).

To produce TaqPIN, which incorporates all nucleic acid reagents for independent target‐specific reaction, primers, and probes were initially encapsulated into DEs via the aforementioned method. Subsequently, the DE suspension was blended with the prepolymer solution, followed by micro molding on pre‐patterned PDMS and photopolymerization, thus completing the fabrication of TaqPIN microparticles. This microparticle fabrication process based on micro molding is massively parallel and cost‐effective, which can produce hundreds of microparticles in a 2 × 1 cm^2^ mold at a time. To assess the entrapping efficacy of DEs in the hydrogel matrix, FAM‐DNA‐containing DEs were employed. As a result, >90% of the initial fluorescence intensity was retained in the hydrogel even after a rigorous washing process, indicating that DEs and their contents were effectively entrapped within the hydrogel (Figure S6, Supporting Information). Despite the softness of DEs and the porosity of hydrogels, the hydrogel network is likely to be crosslinked around the DEs to stably trap and protect them. The cryogenic scanning electron microscope (cryo‐SEM) image showed that the hydrogel matrix with DEs had large crater‐like spaces in the matrix, which differed from their neighboring pores or the pores of the matrix without DEs (**Figure** **3**a). This discrepancy may be attributable to the evaporation and subsequent shrinkage of the DEs during the sample preparation for cryo‐SEM. Based on the fluorescence measurement and cryo‐SEM observation, it can be concluded that the DEs were physically well trapped in the hydrogel matrix and that their structure was maintained during TaqPIN production and storage.

### 2.5 Hydrogel Matrix Improves Storage and Release of the Reagents in DEs

It is known that soft structures, such as emulsions, are more stable when trapped in a hydrogel network.^[^ ^13^ ^]^ To explore the stability of DE trapped in the hydrogel matrix, both emulsion suspensions and hydrogel microparticles containing DE were centrifuged at 10 000 rpm for 30 s to induce shear stress in the emulsion. FAM‐DNA was loaded into DE for fluorescence measurement, and the fluorescence change before and after centrifugation followed by buffer exchange was measured (Figure 3b). The results showed that DE entrapped in hydrogel microparticles demonstrated greater stability than that in suspension. This was likely due to the inherent flow resistance within the hydrogel matrix, which stalls the flow inside the matrix, thus suppressing direct shear forces on DE.

In addition to the stability, the release of FAM‐DNA from DEs trapped in the hydrogel matrix upon heating was assessed. The composition of surfactants for DEs was set to 2 and 8 CMC for oleophilic surfactants and 37.5 and 50.0 CMC for hydrophilic surfactants, respectively, based on the above optimization of DE composition. Ultimately, 8 CMC oleophilic surfactant and 37.5 CMC hydrophilic surfactant were selected as the optimal concentrations for the surfactants, exhibiting an encapsulation concentration of 5.6 µmol L^−1^, a retention efficiency of 89.6% at 55 °C (temperature for RT), and a release efficiency of 47.1% at 95 °C (temperature for PCR) (Figure 3c,d). Compared with the DE suspension, the release efficiency was significantly increased in the form of DE trapped in a hydrogel matrix.

To scrutinize what happens to the DEs as the temperatures rise, an observable‐sized DE suspension was produced and the changes in DE were observed as the temperature was gradually increased. The thermodynamic instability of DE caused by heating was found to be resolved primarily by the merger between DEs and the expulsion of W1 to the external phase (Figure S7, Supporting Information). When the DEs were physically trapped in the hydrogel matrix, their mobility was severely restricted, making it virtually impossible for them to encounter each other; therefore, merging between DEs was unlikely to occur. Consequently, W1 expulsion would be the dominant factor in resolving the increased thermodynamic instability caused by heating, resulting in the release of the reagents. Therefore, we believe that the trapping of DEs within a hydrogel matrix not only improves storage stability but also facilitates the more effective and timely release of the reagents.

### 2.6 RT‐qPCR with TaqPIN

**Figure 4:**
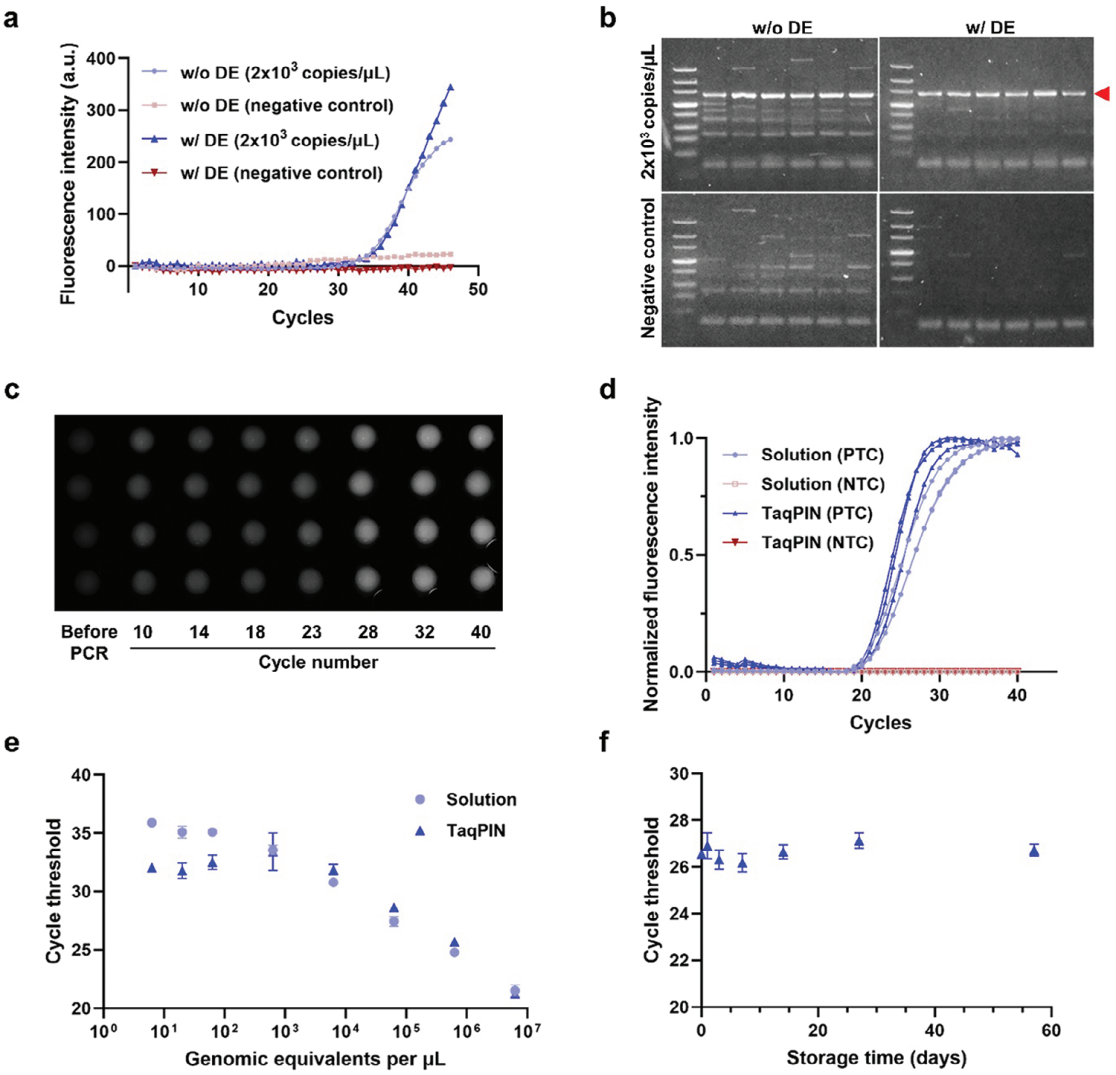
Evaluation of DE as a carrier of PCR reagents. a) A comparison of amplification curves obtained from conventional RT‐qPCR (w/o DE) and RT‐qPCR with DEs (w/DE). No amplification signal was observed in negative controls. b) An analysis of byproducts generated from conventional RT‐qPCR or RT‐qPCR with DE using agarose gel electrophoresis, demonstrating a reduction in byproducts when DE was employed as a reagent carrier. This may be attributed to the suppression of non‐target amplification resulting from the encapsulation of the PCR primer by DE. A red arrow indicates the target bands. c) Sequential fluorescence images during TaqPIN RT‐qPCR showed an increase in fluorescence intensity within the microparticles as the amplification proceeded. Four independent TaqPIN RT‐qPCRs were performed. d) Amplification curves of conventional RT‐qPCR (solution) and TaqPIN RT‐qPCR. e) Sensitivity of RT‐qPCR with TaqPINs. f) Storage stability of TaqPINs (n = 3). The Ct values of TaqPIN RT‐qPCR showed a Ct value variation of less than 1 over 58 days (n = 3). PTC, positive template control; NTC, no template control.

Before fully implementing the TaqPIN assay, solution‐phase RT‐qPCR with DEs was conducted to directly ascertain the specific effect of DEs during RT‐qPCR. PCR primers and probes targeting the nucleocapsid (N) gene of SARS‐CoV‐2 were introduced as encapsulated in DEs, while RT primers were directly mixed with the RT‐qPCR mastermix. A one‐step RT‐qPCR was carried out using synthetic N gene RNA templates (2 × 10^3^ copies/µL) spiked in total RNA (50 ng) isolated from the human embryonic kidney (HEK) 293T cell line to simulate complex clinical samples (positive control, PC), and the results were compared with those obtained using an assay where all primers and probes were provided as a mixture with the mastermix, as in conventional RT‐qPCR. The results obtained with the RNA template showed a minor difference in cycle threshold (Ct) values between the RT‐qPCR with DEs (Ct = 34.8) and without DEs (Ct = 34.2), while both showed no signal from the negative control (NC) (**Figure** **4**a). This indicates that the DEs effectively released the primers and probes promptly for the amplification process. Gel electrophoresis of the products yielded information relevant to the byproducts generated during the RT‐PCR (Figure 4b). It is noteworthy that, despite the higher RT temperature than usual, the reaction with DEs showed a concrete suppression of byproduct generation compared to the reaction without DEs, in both PC and NC. If RT had been conducted at a lower temperature (≈42 °C), as is commonly done, it could potentially result in the formation of more byproducts during RT, particularly in complex samples with abundant background nucleic acids. The storage of PCR primers in DEs during RT circumvents the non‐specific reactions between hetero‐primers or PCR primers and non‐target nucleic acids, in contrast to a solution‐phase assay where both primers were reactive in RT. This target‐specific RT process with DEs is of importance because non‐target amplification may consume reagents such as primers, dNTPs, and Taq polymerase in the PCR step, which should be used for target amplification. Although probe‐based assays can exclude non‐specific signals resulting from byproducts, the unnecessary consumption of the reagents may lead to the failure of the detection of less‐abundant targets. As anticipated, the RT‐qPCR conducted with 200 copies of synthetic N gene RNA spiked in total RNA (50 ng) revealed that only the reaction containing DEs presented a target‐specific signal (Figure S8, Supporting Information). The solution‐phase RT‐qPCR with DEs demonstrated that thermo‐responsive DEs showed effective carrier properties for reagents in RT‐qPCR, integrating the RT and PCR processes seamlessly, and thus suppressing the non‐specific reaction during RT‐PCR.

Based on the successful solution‐phase RT‐qPCR with DEs, RT‐qPCR in the form of TaqPIN was carried out. As the amplification proceeded, the fluorescence signal of the microparticles became uniformly distinct, indicating that the fluorescence reporters hydrolyzed by the polymerase had been stably accumulated within the microparticles (Figure 4c). Using an equivalent concentration of viral RNA (6.02 × 10^6^ genome equivalent (GE)/µL), TaqPIN RT‐qPCR yielded an equivalent Ct value to the solution‐phase RT‐qPCR, thereby affirming the comparable detection efficacy of the TaqPIN assay even with a natural form of viral RNA (Figure 4d). To see the detailed performance of the TaqPIN RT‐qPCR, a TaqPIN RT‐qPCR with a 10‐fold serial dilution was performed with viral RNA. A consistent gap in Ct values was observed within the range of sample concentrations from 6.02 × 10^6^ to 6.02 × 10^2^ GE/µL, which is similar to that observed in solution‐phase RT‐qPCR (Figure 4e). Based on these serial dilution data, the amplification efficiency of TaqPIN RT‐qPCR was calculated to be 93.8%. At a sample concentration of 6.02 GE µL^−1^, due to the limited amount of RNA, not all particles produced a fluorescent signal (two of three TaqPINs presented fluorescence signals) (Figure S9, Supporting Information). TaqPIN RT‐qPCR also demonstrated a limit of detection of 20 GE µL^−1^, with all TaqPINs displaying fluorescence signals. The results demonstrate that TaqPIN provides primers and probes for the consistent and accurate amplification of target nuclei throughout the PCR process. Furthermore, the amplification performance of the TaqPIN was maintained for over eight weeks when stored at 4 °C, confirming that DEs enable the effective storage and release of primers and probes even in polymer networks (Figure 4f).

### 2.7 Multiplexed Panel for SARS‐CoV‐2 Variant Discrimination

**Figure 5:**
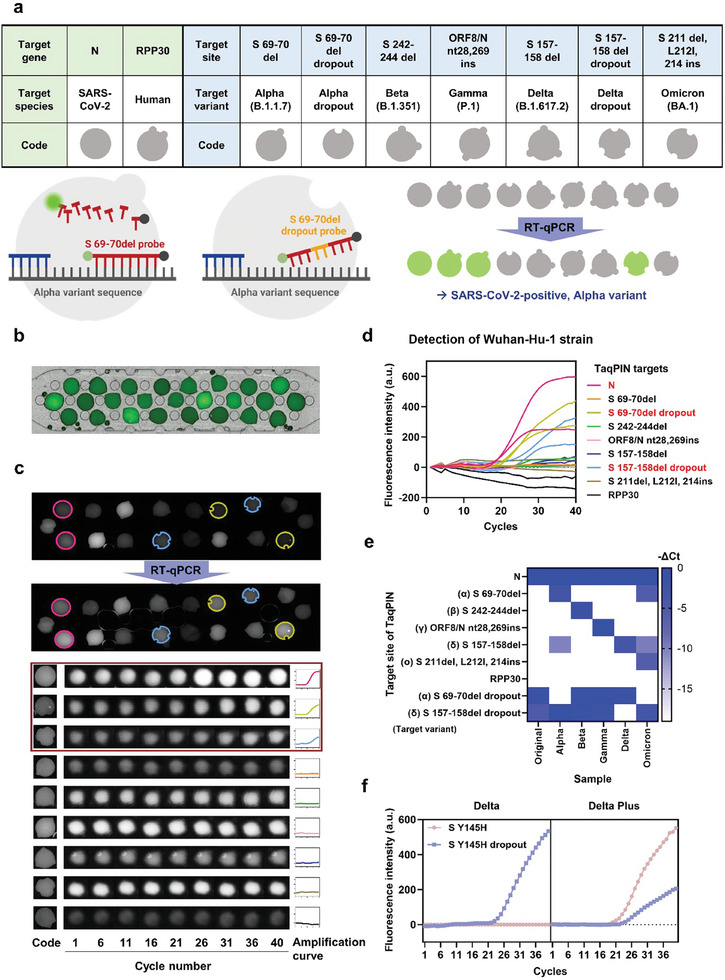
Discrimination of SARS‐CoV‐2 strains using the TaqPIN array. a) TaqPIN RT‐qPCR panel for discriminating six SARS‐CoV‐2 strains. A table presents target sites, viral strains, and distinguishable outer slit code of TaqPIN. The working principle of dropout probe‐embedded TaqPIN, confirming the absence of target mutation, is described. b) A representative image of 25 TaqPIN arrays on a chip. c) The result of multiplexed TaqPIN RT‐qPCR with Wuhan‐Hu‐1 strain. Six out of eighteen TaqPINs showing an increase in fluorescence are highlighted by colored rims. Fluorescence images of individual TaqPINs showed three TaqPINs (targeting the N gene, S 69–70 del dropout, and S 157–158 del dropout) marked with a magenta‐colored box, displaying increases in brightness according to PCR cycle progression. The fluorescence images of TaqPIN on the left side were taken under a fluorescence microscope right before RT‐qPCR, while the rest of the images were obtained on the RT‐qPCR instrument during PCR. The amplification curve for each TaqPIN is displayed to the right of the image sequences. d) The amplification curves of the 18‐plex TaqPIN assay for targeting the Wuhan‐Hu‐1 strain. Targets of TaqPINs showing an increase in fluorescence are highlighted in red. e) Heat map of the results of multiplexed assays with viral RNA. The ΔCt values were calculated by subtracting the Ct value of the N gene from each Ct value and multiplying by −1. f) Discrimination of Delta variant (left) and Delta Plus variant (right) using TaqPINs targeting S Y145H and TaqPINs targeting S Y145H dropout (n = 3).

By assembling several TaqPINs prepared as described above, a multiplexed assay can be conducted with ease and efficiency. As a practical application, we first aimed to simultaneously distinguish the Wuhan‐Hu‐1 strain of SARS‐CoV‐2 and its five variants, including Alpha (B.1.1.7), Beta (B.1.351), Gamma (P.1), Delta (B.1.617.2), and Omicron (BA.1). These variants were designated as variants of concern (VOCs) by the World Health Organization (WHO) during the pandemic.^[^ ^14^ ^]^ Each variant has known mutations that are variant‐specific, for instance, a deletion at amino acid positions 69 and 70 of the spike (S) gene for Alpha (S 69–70 del), a deletion at amino acid positions 242, 243, and 244 of the S gene for Beta (S 242–244 del), insertion of 4 nt after the genomic position 28269 at the intergenic region between open reading frame‐8 and N gene for Gamma (ORF8/N 28,269ins), a deletion at amino acid positions 157 and 158 of the S gene for Delta (S 157–158 del), and deletion of 3nt at genomic positions 22194 and an insertion of 9nt after genomic position 22204 at the S gene for Omicron (S 211 del, L212I, 214ins).^[^ ^15^, ^16^, ^17^ ^]^ Each of the above mutations was selected as a target region for each variant detection, with all showing a difference of 4 to 12 nucleotides from their pre‐existing strains (**Figure** **5**a). In addition, the N gene was selected as a diagnostic marker for SARS‐CoV‐2 regardless of variant types, and the human ribonuclease P/MRP subunit p30 (RPP30) gene was used as an internal control to see the provenance of the clinical sample from the human as well as the assay fidelity, primers and probes for the detection of each target region were validated through conventional solution‐phase RT‐qPCR before the introduction to the TaqPIN assay (Table S3, Supporting Information).

Multiplexed assay leveraging TaqPINs offers remarkable flexibility, as the combination of the particles can be altered as needed. We further utilized this flexibility to clarify the signals. In cases where the primary TaqPIN for a variant exhibited non‐specific signals, the addition of a paired comparative particle enabled the verification of this signal and the assurance of accurate results. In practice, the TaqPINs for dropout sequences have been designed with probes recognizing the wild‐type sequence before the occurrence of mutations at the target site. Subsequently, the resulting dropout TaqPINs are included in the multiplexed assay configuration. Given that non‐specific signals are generally weaker than target‐specific signals,^[^ ^18^ ^]^ we measured target and dropout signals simultaneously from the same sample, enabling comparative analysis for precise distinction, as illustrated in Figure 5a. In this study, we employed this approach to analyze the Alpha and Delta variants of SARS‐CoV‐2 mutations (S 69–70 del and S 157–158 del).

Including the TaqPINs for dropouts, nine TaqPIN microparticles corresponding to seven target sites were prepared, with each TaqPIN having a unique code for easy identification of its target gene (Figure 5a). A set of TaqPINs was then aligned in a single‐chamber plastic chip. The chip was designed to accommodate up to 25 microparticles and was equipped with a post array to streamline the alignment of these particles (Figure 5b; Figure S10, Supporting Information). We conducted multiplexed RT‐qPCR using an array of 18 TaqPINs (two of each TaqPIN) to detect viral RNAs extracted from the Wuhan‐Hu‐1 strain of SARS‐CoV‐2. After the chip was filled with the master mix that was mixed with viral RNA only, oil was injected to isolate each TaqPIN. The chip‐based RT‐qPCR was performed using a planar thermal cycler, and fluorescence images of the array were recorded at the end of each annealing and extension step to track and chart the fluorescence intensity change of each TaqPIN. As shown in Figure 5c, the brightness of the fluorescence images of the three types of TaqPINs (targeting N gene, S 69–70 del dropout, and S 157–158 del dropout) increased as the PCR progressed, while the others did not. The collective results of the 18 particles pointed to the Wuhan‐Hu‐1 strain, in agreement with the sample provided. The nine pairs of particles, each consisting of two identical TaqPINs, gave matching results in each pair (Figure 5c,d). This helped to ensure that the signal from each microparticle was consistent.

To validate the selectivity of TaqPIN‐based multiplexed RT‐qPCR, the assays were performed with viral RNAs extracted from five distinct variants of SARS‐CoV‐2 including Alpha, Beta, Gamma, Delta, and Omicron (Figure S11a–e, Supporting Information). The Ct values derived from nine different TaqPINs within a single assay were normalized against the Ct value derived from the TaqPIN targeting the N gene to generate a heatmap (Figure 5e). The microparticles corresponding to each viral RNA exhibited distinct ΔCt values (Ct(target) – Ct(N gene)), enabling the clear differentiation of SARS‐CoV‐2 variants, except for S 157–158 del for the Delta variant.

**Figure 6:**
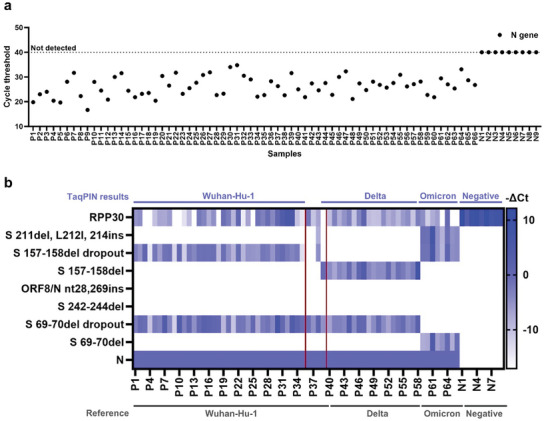
SARS‐CoV‐2 diagnosis and viral strain discrimination of clinical samples using TaqPIN array. a) SARS‐CoV‐2 diagnosis in 75 nasopharyngeal swab samples. The Ct values of TaqPINs targeting the N gene were plotted. b) Heat map of viral strain discrimination. TaqPIN RT‐qPCR results from 66 positive samples were analyzed. The Ct values of TaqPINs were normalized by subtracting the Ct values of the TaqPIN targeting the N gene and multiplying the result by −1. For TaqPINs that showed no fluorescence amplification signal, a Ct value of 40 was assigned for calculation. The SARS‐CoV‐2 strains identified by multiplexed TaqPIN RT‐qPCR are labeled at the top of the heat map. Four discrepancies, compared to the sequencing results shown at the bottom of the heat map, are highlighted in a magenta‐colored box.

The use of comparative microparticle pairs of target/dropout was highly effective in determining this Delta variant. The TaqPIN of the Delta variant at S 157–158 del showed weak false‐positive signals for Alpha and Omicron samples (Figure 5e; Figure S11f, Supporting Information). However, the counterpart TaqPIN, dropout of S 157–158 del, exhibited clear positive signals, and by comparing the signals of the two pairs of microparticles, the weak signals for Alpha and Omicron samples in the TaqPIN of S 157–158 del were definitively determined as “not‐detected” calls. If there had not been a clear contrasting signal in the dropout of S 157–158 del, an incorrect conclusion could have been drawn that the Alpha or Omicron samples contained trace amounts of the Delta variant virus. Thus, the target‐dropout comparative strategy can filter out and distinguish false‐positive signals by using a pair of TaqPIN microparticles to recognize each target and dropout. Consequently, using a straightforward 40‐min RT‐qPCR, we achieved the variant detection accuracy of 93.9% in the analysis of 75 clinical samples; A detailed account of the results will be provided in Figure 6.

### 2.8 Single Nucleotide Discrimination with LNA Probe

The AY.4.2 strain is a sublineage of the Delta variant, which was discovered after the widespread dissemination of the Delta variant. The AY.4.2 strain can be identified by a single base change resulting in an amino acid substitution from tyrosine to histidine at position 145 of the S gene (S Y145H) from the Delta variant (Figure S12, Supporting Information). Despite differing by only one base, its transmission capability was significantly higher, earning it the nickname “Delta Plus.” To distinguish such subtle mutations of a single nucleotide, it is necessary to finely adjust the PCR assay's temperature and the composition of the mastermix to ensure effective discrimination even with minimal differences. This makes it very difficult to conduct multiplexed assays simultaneously.^[^ ^19^ ^]^ In such cases, artificial probes are sometimes used to enhance differentiation accuracy.^[^ ^20^ ^]^ Here, a probe capable of distinguishing Delta Plus from the Delta variant was designed to include locked nucleic acids (LNAs) (Figure S13a, Supporting Information). For the S Y145H mutation, the melting temperature difference of the DNA probe was calculated to be 1.8 °C, but this widened to 12 °C when using the LNA probe, as confirmed by the signal difference observed in PCR experiments (Figure S13b, Supporting Information). The TaqPIN assay results indicated that the S Y145H and its corresponding dropout exhibited clear positive signals for the Delta Plus and Delta variants, respectively (Figure 5f). With the aid of LNA probes, the S Y145H probe provided a clear “not‐detected” signal for the Delta variant without any cross‐reactivity. In contrast, the S Y145H dropout showed a marginally improved but insufficient signal difference with the LNA probe, with the maximum melting temperature difference calculated to be only 3 °C (Figure S13c, Supporting Information). Consequently, the TaqPIN for the S Y145H dropout showed a false‐positive signal for Delta Plus samples. This is an unavoidable outcome, as the annealing temperature for each PCR cannot be perfectly optimized in multiplexed analysis.

The comparison of the target/dropout pair can also make signal interpretation more reliable in this case. For the Delta Plus variant in Figure 5f, a TaqPIN of S Y145H had a considerably more intense signal than the one of S Y145H dropout, which confirms ‘S Y145H is detected’. Notably, all conditions of the nine‐plex assay could remain unchanged, with the addition of only a pair of TaqPIN microparticles, allowing for the simultaneous implementation of all assays, including those for single nucleotide variations. In the event of widespread viral dissemination, keeping up with viral mutations in diagnostics without delay is crucial for maintaining stable quarantine measures. The flexibility of the TaqPIN array allows for the addition of only a few new TaqPINs while maintaining the original assay performance in multiplexed PCR analysis, making it a suitable advantage for rapid emergency response.

### 2.9 Clinical Test

We finally investigated the clinical utility of the TaqPIN panel for discriminating various strains of SARS‐CoV‐2. A total of 75 nasopharyngeal swab samples were collected, comprising 66 from COVID‐19 patients and 9 from healthy donors. The TaqPIN RT‐qPCR demonstrated exceptional sensitivity and specificity at 100% for both positive and negative sample determinations in our limited cohort, even though over 20% of the samples had Ct values equal to or more than 30, which are typically considered challenging (**Figure** **6**a and Table S4, Supporting Information).^[^ ^21^ ^]^ Specifically, the TaqPINs targeting the N gene consistently exhibited robust amplification signals in positive samples, while negative samples exhibited no signal amplification. Moreover, in terms of variant determination, the TaqPIN RT‐qPCR displayed a noteworthy 93.9% agreement (62 out of 66) with Sanger sequencing, emphasizing its fidelity in accurately identifying variants (Figure 6b). Our findings demonstrate the remarkable accuracy of the TaqPIN RT‐qPCR in detecting SARS‐CoV‐2 and its various strains. These results emphasize the critical role of a comprehensive assessment of mutations at multiple loci for accurate variant determination. We believe that TaqPIN‐based multiplexed panel tests will effectively mitigate the difficulties arising from slightly different optimal conditions between assays, ultimately leading to more precise and comprehensive diagnostic outcomes.

## 3 Discussion

Various diagnostic techniques have been suggested to simultaneously detect and differentiate multiple SARS‐CoV‐2 genetic mutations associated with distinct viral strains.^[^ ^22^, ^23^ ^]^ In general, these assays rely on nucleic acid amplification tests (NAATs), such as RT‐qPCR, loop‐mediated isothermal amplification (LAMP), clustered regularly interspaced short palindromic repeats (CRISPR)‐based detection (Table S5, Supporting Information).^[^ ^23^, ^24^ ^]^ Other technologies besides PCR also have their unique advantages, offering promising new solutions that are suitable for specific purposes and environments. However, despite their purported high performance, they lack the assay design protocols and know‐how accumulated over time that PCR has. Therefore, when a new target gene emerges, it needs to be developed from the ground up through multiple iterations to improve performance.

On the contrary, the TaqPIN assay uses identical primer and probe sequences, which are validated in solution‐phase RT‐PCR without further sequence optimization. In the event of a new emerging infectious disease, the corresponding PCR assays are set up and announced by various specialized organizations within a few days. With that information, a TaqPIN assay can be immediately developed. Furthermore, adding the new microparticles to existing diagnostic panels completes updating multiplexed assays with no need for additional optimization.

For a multiplexed assay, instead of using different colored probes, the individual reactors are isolated in the hydrogel microparticles, arrayed in a single chip, and their fluorescence signals are monitored even with one‐color fluorescence optics. Since the reactions are physically isolated, the non‐specific signals produced by promiscuous reactions among multiple primers and probes are restrained. This enables the expansion of multiplex detection capability to detect well over 10 species, up to dozens simultaneously, by adding more microparticles without the risk of interference between reactions. The TaqPIN assay also allows for accurate gene expression quantification in a multiplexed setting by recording fluorescence intensity at each cycle during PCR. This study only presents the viral variant distinction as a timely example, but it can be extended to a variety of multiplexed assays. For instance, it can be utilized for syndromic multiplex testing by modulating the combination of microparticles or for multiplexed transcriptomic profiling to simultaneously read quantitative changes. This TaqPIN assay will provide a simple but reliable and practical tool for the molecular diagnostics of multiple targets.

## 4 Conclusion

In conclusion, we have utilized W/O/W DE to carry and deliver nucleic acid reagents essential for target‐specific RT‐PCR, specifically primers and probes. The unique features of DE were pivotal in encapsulating and triggering the release of these reagents. The incorporation of DE with target‐specific reagents into the hydrogel matrix greatly improved its stability and release, owing to the porous yet cohesive structure of the hydrogel. This facilitated the seamless connection of the two enzymatic reactions, reverse transcriptase and DNA polymerase, while minimizing their interference in a hydrogel microparticle named TaqPIN. The versatility of TaqPINs enabled multiplexed assays owing to the pre‐contained target‐specific reagents within the microparticles. For more precise mutation detection, strategies like target‐dropout comparison or the use of highly selective modified nucleic acid probes were easily integrated into the TaqPIN assay.

Fundamentally, DE in the hydrogel network serves as a stable carrier that can store hydrophilic substances and release them at desired times through temperature control. It can also be used to implement sequential operations in enzyme reactions or chemical reactions with different operating temperatures. Consequently, the technology is highly versatile with significant potential for new applications requiring timely release for the reaction. It is therefore expected that various studies investigating the effects of varying the working temperature or loading different substances will be conducted in the future.

## 5 Experimental Section

### Preparation of Double Emulsions

An LMPA solution as a gelling agent was prepared by dissolving 25 mg of LMPA powder (Promega, USA) into 1 mL of phosphate‐buffered saline (PBS, Lonza, USA) at 100 °C for 20 min, with vortexing every 5 min. The internal water phase consisting of nucleic acid‐based reagent (4 µL, 1 mmol L^−1^, IDT, USA) and the LMPA solution (16 µL, 2.5 wt%) was pipetted into the continuous oil phase (200 µL) consisting of HFE‐7500 (3 m, USA) and 008‐fluoro‐surfactant (0.4 wt%, RAN Biotechnologies, USA). Tip sonication (Q125 sonicator, Qsonica, USA) was operated at 40% amplitude for 45 sec to generate a single water‐in‐oil (W1/O) emulsion. After incubating the product at 4 °C for 10 min to stabilize the single emulsion by reducing the heat generated during the sonication process and solidifying LMPA, the emulsion was separated from the oil phase through centrifugation at 13 000 rpm for 40 s. After the oil phase was maximally removed, the external water phase (1 mL) consisting of PBS with Tween20 (0.25 vol%, Sigma–Aldrich, USA) (PBST) was introduced. Another tip sonication was performed at 40% amplitude for 10 s to disperse single emulsions in the continuous water phase and generate W1/O/W2 double emulsions. To separate the emulsion from the water phase, centrifugation was conducted at 10000 rpm for 10 s, and the supernatant was removed. The final DE was re‐suspended with 35 µL of PBST.

### Characterization of DEs

The size of DEs was measured using DLS (Zetasizer Nano ZS instrument, Malvern Co., UK). DEs were 10‐fold diluted with PBST to make them the proper concentration for the analysis. Measurements were conducted with a scattering angle of 90° at a temperature of 25 °C. Data from triplicate measurements were averaged for each sample, and the DLS raw data were processed using the regularization algorithm provided by the software (Zetasizer Nano software).

The structure of DEs was characterized using cryogenic transmission electron microscopy (cryo‐TEM). A 3 µL of DE solution was deposited on a TEM grid for 30 s. The excess solution was blotted out with filter paper for 3 s, and the sample was rapidly plunged into liquid ethane precooled with liquid nitrogen using a vitrobot FP 5350/60 (FEI, Netherlands) at 22 °C and 100% humidity. The vitrified samples were then transferred to a Gatan 626 cryo‐holder (Gatan, USA) and imaged using a cryo‐TEM (Tecnai F20 G2, FEI).

To assess the encapsulation and release efficiencies of DEs, DEs encapsulating fluorescein FAM‐DNA with an arbitrary sequence (5ʹ‐GGT CGA GGG TGG CTA CGG CTG AAC T[FAM]‐3ʹ) was prepared. Fluorescence measurements of the DEs were performed in triplicate using a fluorescence spectrometer (FLUOstar Omega, BMG LABTECH, Germany). The DEs were 10‐fold diluted with PBST and dispensed in 80 µL into a 96‐well plate. The concentration of DNA encapsulated in the DEs was estimated by fitting the measured fluorescence intensity to a standard curve generated from the fluorescence intensities of solutions containing various known quantities of FAM‐DNA (0, 1, 2.5, 5, 10, and 20 µmol L^−1^).

To estimate DE release, 20 µL of DEs encapsulating FAM‐DNA was incubated at 55 and 95 °C for 10 min. Subsequently, the DEs were sedimented by centrifugation at 10000 rpm for 10 min, and 10 µL of the supernatants were collected. Fluorescence intensities of 10‐fold dilutions of the supernatants with PBST were measured using fluorescence spectrometry. The concentration of DNA released from the DEs was estimated by fitting the measured fluorescence intensity to a standard curve. The release efficiency was calculated as the ratio of the amount of DNA released to the amount of DNA encapsulated.

### Preparation of TaqPIN Microparticles

A photoinitiator aqueous solution was prepared by dissolving 0.2 g of diphenyl(2,4,6‐trimethylbenzoyl)phosphine oxide (TPO) based nanoparticle photoinitiator (Sigma–Aldrich) in 1g of water. A photocurable pre‐polymer solution was prepared by mixing poly(ethylene glycol) diacrylate (20%, PEGDA Mn 700, Sigma‐Aldrich), PEG (40%, Mn 600, Sigma–Aldrich), the photoinitiator aqueous solution (5%), DE suspension containing PCR primers (17.5%), and TaqMan probes (17.5%) through vortexing. PEG served as a porogen, preserving pores to facilitate mass transfer, thereby supporting the reaction within TaqPIN during RT‐qPCR. The solution for a TaqPIN was finalized by mixing the pre‐polymer solution and acrydite‐modified RT primers (1 mmol L^−1^, IDT) with a volume ratio of 20:1. The solution for a TaqPIN was spotted on the pre‐patterned PDMS mold, which was treated with oxygen plasma in advance to enhance pre‐polymer solution spreading. PDMS mold was a circular well type with different outer slit codes, which produces disk‐shaped microparticles (650 µm in diameter and 330 µm in height) with slit codes after photo‐curing. The excess pre‐polymer solution over the mold was removed by scraping off the surface of the PDMS mold with a spatula and pressing the mold with a sliding glass. The pre‐polymer filled into the PDMS mold was cured by the exposure of 50 mJ of UV light. Cured TaqPIN was thoroughly washed by PBST to remove the porogen and unbound primers and stored in the PBST until use.

### Characterization of TaqPIN

The dimension and fluorescence intensity of TaqPIN were measured using fluorescence microscopy (Celena X High Content Imaging System, Logos Biosystems, Korea). To evaluate encapsulation efficiency, TaqPINs were fabricated with DEs containing FAM‐DNAs. To build a standard curve, 10 TaqPINs incorporating DEs without FAM‐DNA were loaded onto each microfluidic chip. Serial dilutions of FAM‐DNA solutions (10, 5, 2.5, 1, and 0 µm) were injected into each chip. Solutions adjacent to TaqPINs were removed, and the TaqPINs were isolated with silicone oil. Fluorescence images of TaqPINs were captured using Celena X, and their fluorescence intensities were analyzed with ImageJ (public domain software from the National Institutes of Health; http://imagej.nih.gov/ij/). The encapsulation efficiency of the TaqPIN was evaluated by correlating fluorescence intensity with the equivalent DNA concentration. TaqPINs made with FAM‐DNA‐containing DEs were transferred to 1.7 mL microcentrifuge tubes and incubated at 55 °C to assess retention efficiency and at 95 °C to evaluate release efficiency, both for 10 min. After cooling on ice, the TaqPINs were loaded onto chips, and imaged by Celena X. The fluorescence intensities of the TaqPINs were analyzed with ImageJ. Retention and release efficiencies were calculated by fitting the fluorescence intensity to the standard curve.

### Cryo‐SEM Measurement

Cryo‐SEM measurements were carried out using the Quanta 3D FEG (FEI) with the ALTO 2500 cryo‐transfer system (Gatan). The sample, securely fixed within the SEM holder, was transferred to the ALTO 2500 preparation chamber for further processing. Freeze‐fracturing of the sample was executed utilizing the blade on the ALTO 2500 manipulator. Subsequently, the sample underwent etching at −95 °C for ≈5–10 min. Following the etching process, a coating of Au/Pd metal was applied to the sample surface using a target in the preparation chamber. The prepared sample was then transferred to the SEM chamber equipped with a cryo‐stage, and image observation was conducted in a temperature range of −160 to −140 °C.

### Virus Samples

The genomic RNA of the original Wuhan‐Hu‐1 strain of SARS‐CoV‐2 (NCCP 43326 and NCCP 43327), B.1.1.7 (Alpha variant, NCCP 43381), B.1.351 (Beta variant, NCCP 43382), P.1 (Gamma variant, NCCP 43388), B.1.617.2 (Delta variant, NCCP 43390), and BA.1 (Omicron variant, NCCP 43408) were obtained from the National Culture Collection for Pathogens (NCCP, Korea). Viral RNA was extracted from virus cultures derived from respiratory specimens confirmed to be positive for COVID‐19 in Korea.

### TaqPIN RT‐qPCR

The PCR cocktail was prepared by mixing 5 µL of 2X OneStep Master Mix (TaqMan, RNA) (MicoBiomed, Korea), 1–5 µL of template, and Ambion nuclease‐free water (Invitrogen, USA) to complete 10 µL in total. The cocktail was mixed with TaqPINs by low‐speed vortexing for 1 min, followed by loading them onto a plastic PCR chip. The film was used to finalize a microfluidic chip by sealing the bottom of the chip. Silicone oil was injected into the chip for isolation of the TaqPINs. TaqPINs‐based one‐step RT‐qPCR was conducted using a Veri‐Q PCR 204 (Mico BioMed). The thermocycling condition for RT‐qPCR was as follows: reverse transcription at 55 °C for 8 min and pre‐denaturation at 95 °C for 10 s, followed by 40 cycles of denaturation at 95 °C for 5 s and annealing and extension at 55 °C for 30 s. At the end of each PCR cycle, the fluorescence intensities of each TaqPIN were measured and recorded. For multiplexed assays, nine TaqPINs, each targeting a different gene, were loaded onto the chip. One to three TaqPINs for each target were arrayed, and the PCR cocktail was prepared in a total volume of 16 µL. The rest of the process was the same as described above.

### Clinical Samples

RNA was extracted from 75 nasopharyngeal swab samples obtained from 66 coronavirus disease 2019 (COVID‐19) patients, and 9 individuals tested negative for the virus. The collection and utilization of samples were conducted in accordance with protocols approved by the Institutional Review Board (IRB) of Seoul Asan Medical Center (Approval No. S2022‐2265‐0003). The RNA extraction process employed a Starlet instrument with the STARMag 96 × 4 Universal Cartridge Kit. To encompass diverse viral strains, samples isolated during four distinct time periods (November 2020, May 2021, November 2021, and January 2022) within the COVID‐19 pandemic were included in the study. To verify the sequence of the target site, the PCR product underwent agarose gel electrophoresis, and the specific gel band corresponding to the target was excised. The excised gel slice was then sent to Bioneer (Korea) for Sanger sequencing.

## Conflict of Interest

The authors declare no conflict of interest.

## Supporting information



Supporting Information

## Data Availability

The data that support the findings of this study are available from the corresponding author upon reasonable request.

## References

[1] D. J. McClements , E. A. Decker , J. Weiss , J. Food Sci. 2007, 72, R109. 17995616 10.1111/j.1750-3841.2007.00507.x

[2] D. J. McClements , Y. Li , Adv. Colloid Interface Sci. 2010, 159, 213. 20638649 10.1016/j.cis.2010.06.010

[3] a) M. Iqbal , N. Zafar , H. Fessi , A. Elaissari , Int. J. Pharmaceut. 2015, 496, 173; 10.1016/j.ijpharm.2015.10.057 26522982

[4] a) D. T. Chong , X. S. Liu , H. J. Ma , G. Y. Huang , Y. L. Han , X. Y. Cui , J. J. Yan , F. Xu , Microfluid. Nanofluid. 2015, 19, 1071;

[5] a) B. J. Hindson , K. D. Ness , D. A. Masquelier , P. Belgrader , N. J. Heredia , A. J. Makarewicz , I. J. Bright , M. Y. Lucero , A. L. Hiddessen , T. C. Legler , T. K. Kitano , M. R. Hodel , J. F. Petersen , P. W. Wyatt , E. R. Steenblock , P. H. Shah , L. J. Bousse , C. B. Troup , J. C. Mellen , D. K. Wittmann , N. G. Erndt , T. H. Cauley , R. T. Koehler , A. P. So , S. Dube , K. A. Rose , L. Montesclaros , S. L. Wang , D. P. Stumbo , S. P. Hodges , et al., Anal. Chem. 2011, 83, 8604; 22035192 10.1021/ac202028g PMC3216358

[6] a) E. Z. Macosko , A. Basu , R. Satija , J. Nemesh , K. Shekhar , M. Goldman , I. Tirosh , A. R. Bialas , N. Kamitaki , E. M. Martersteck , J. J. Trombetta , D. A. Weitz , J. R. Sanes , A. K. Shalek , A. Regev , S. A. McCarroll , Cell 2015, 161, 1202; 26000488 10.1016/j.cell.2015.05.002 PMC4481139

[7] D. J. Collins , A. Neild , A. deMello , A. Q. Liu , Y. Ai , Lab Chip 2015, 15, 3439. 26226550 10.1039/c5lc00614g

[8] a) S. Acter , M. L. P. Vidallon , J. P. King , B. M. Teo , R. F. Tabor , J. Mater. Chem. B 2021, 9, 8962; 34569589 10.1039/d1tb01796a

[9] a) A. P. Esser‐Kahn , S. A. Odom , N. R. Sottos , S. R. White , J. S. Moore , Macromolecules 2011, 44, 5539;

[10] D. J. McClements , Curr. Opin. Colloid In. 2004, 9, 305.

[11] a) R. Evans , G. Dal Poggetto , M. Nilsson , G. A. Morris , Anal. Chem. 2018, 90, 3987; 29481057 10.1021/acs.analchem.7b05032

[12] M. C. Lee , C. Tan , R. Ravanfar , A. Abbaspourrad , ACS Appl. Mater. Inter. 2019, 11, 26433. 10.1021/acsami.9b05089 31245993

[13] a) J. Y. Wen , Y. Zhang , H. N. Jin , X. N. Sui , L. Z. Jiang , J. Agr. Food Chem. 2020, 68, 9796; 32786850 10.1021/acs.jafc.0c03586

[14] a) L. Mautner , M. Hoyos , A. Dangel , C. Berger , A. Ehrhardt , A. Baiker , Virol. J. 2022, 19, 76; 35473640 10.1186/s12985-022-01802-5 PMC9038516

[15] K. Yaniv , E. Ozer , M. Shagan , S. Lakkakula , N. Plotkin , N. S. Bhandarkar , A. Kushmaro , Environ. Res. 2021, 201, 111653. 34245731 10.1016/j.envres.2021.111653 PMC8262398

[16] K. Yaniv , E. Ozer , Y. Lewis , A. Kushmaro , Water Res. 2021, 207, 117808. 34753092 10.1016/j.watres.2021.117808 PMC8551083

[17] M. Gerdol , K. Dishnica , A. Giorgetti , Virus Res. 2022, 310, 198674. 35021068 10.1016/j.virusres.2022.198674 PMC8743576

[18] R. Nyaruaba , C. Li , C. Mwaliko , M. Mwau , N. Odiwuor , E. Muturi , C. Muema , J. Xiong , J. Li , J. Yu , H. Wei , Expert Rev. Mol. Diagn. 2021, 21, 119. 33380245 10.1080/14737159.2021.1865807 PMC7784781

[19] J. M. Obliosca , S. Y. Cheng , Y. A. Chen , M. F. Llanos , Y. L. Liu , D. M. Imphean , D. R. Bell , J. T. Petty , P. Ren , H. C. Yeh , J. Am. Chem. Soc. 2017, 139, 7110. 28463488 10.1021/jacs.7b03395 PMC5850941

[20] D. A. Braasch , D. R. Corey , Chem. Biol. 2001, 8, 1. 11182314 10.1016/s1074-5521(00)00058-2

[21] V. L. D. Thi , K. Herbst , K. Boerner , M. Meurer , L. P. M. Kremer , D. Kirrmaier , A. Freistaedter , D. Papagiannidis , C. Galmozzi , M. L. Stanifer , S. Boulant , S. Klein , P. Chlanda , D. Khalid , I. B. Miranda , P. Schnitzler , H. G. Kräusslich , M. Knop , S. Anders , Sci. Transl. Med. 2020, 12, eabc7075. 32719001 10.1126/scitranslmed.abc7075 PMC7574920

[22] a) N. L. Welch , M. L. Zhu , C. Hua , J. Weller , M. E. Mirhashemi , T. G. Nguyen , S. Mantena , M. R. Bauer , B. M. Shaw , C. M. Ackerman , S. G. Thakku , M. W. Tse , J. Kehe , M. M. Uwera , J. S. Eversley , D. A. Bielwaski , G. McGrath , J. Braidt , J. Johnson , F. Cerrato , G. K. Moreno , L. A. Krasilnikova , B. A. Petros , G. L. Gionet , E. King , R. C. Huard , S. K. Jalbert , M. L. Cleary , N. A. Fitzgerald , S. B. Gabriel , et al., Nat. Med. 2022, 28, 1083; 35130561 10.1038/s41591-022-01734-1 PMC9117129

[23] a) A. Y. Trick , F. E. Chen , L. B. Chen , P. W. Lee , A. C. Hasnain , H. H. Mostafa , K. C. Carroll , T. H. Wang , Adv. Mater. Technol. 2022, 7, 2101013; 35441089 10.1002/admt.202101013 PMC9011450

[24] A. Y. Trick , F. E. Chen , L. Chen , P. W. Lee , A. C. Hasnain , H. H. Mostafa , K. C. Carroll , T. H. Wang , Adv. Mater. Technol. 2022, 7, 2101013. 35441089 10.1002/admt.202101013 PMC9011450

