# Supplementary material for: Thermally Triggered Double Emulsion‐Integrated Hydrogel Microparticles for Multiplexed Molecular Diagnostics
Source: Adv Sci (Weinh). 2025 Jan 16;12(12):2408158. doi: 10.1002/advs.202408158 (PMC11948052; doi:10.1002/advs.202408158)
Supplement: Supplementary file 1 — Supporting Information [file ADVS-12-2408158-s001.docx]

Supporting Information

Thermally Triggered Double Emulsion-Integrated Hydrogel Microparticles for Multiplexed Molecular Diagnostics

Eui Ju Jeon, Seungwon Jung*, Yoon-ha Jang, Seoyoung Lee, Song-Ee Choi, So Young Jeon, Lankyeong Yoon, Bong Kyun Kim, Tae Jong Kim, Keunyoul Park, Seok Chung, Yong Shin, Sung-Han Kim, Heungsub Sung, Sang Kyung Kim*

**Contents**

**Supporting figures**

1. Oleophilic surfactant screening.
2. Cryo-TEM and cryo-SEM analyses of DEs.
3. Preparation of W1/O single emulsions with varying amounts of oleophilic surfactant.
4. Images of W1/O/W2 DEs prepared with varying amounts of hydrophilic surfactant.
5. Release test of FAM-DNA in DEs during thermal cycling
6. Stability of FAM-modified DNA-encapsulated DEs in hydrogel.
7. Thermal destabilization of micro-sized DEs.
8. Increased sensitivity due to suppression of non-specific amplifications in the reactions containing DEs.
9. Detection of a low-concentration sample (6.2 copies/μL) using TaqPIN array.
10. Multiplexed RT-qPCR detecting Delta variant sample with an array of 25 TaqPINs.
11. Discrimination of SARS-CoV-2 variants using TaqPIN RT-qPCR.
12. LNA probe design for precise discrimination of single nucleotide variation in Delta Plus variant.
13. Discrimination of a single nucleotide variation using LNA probes.

**Supporting tables**

1. DNA encapsulation and release efficiency of DEs as a function of the amount of lipophilic surfactant.
2. DNA encapsulation and release efficiency of DEs as a function of the amount of hydrophilic surfactant.
3. Sequences of primers and probes used for the detection of SARS-CoV-2 variants.
4. Distribution of Ct values of positive samples used for clinical tests.
5. Comparative analysis of the detection methods used for SARS-CoV-2 and its variants.

**Supporting figures**

**
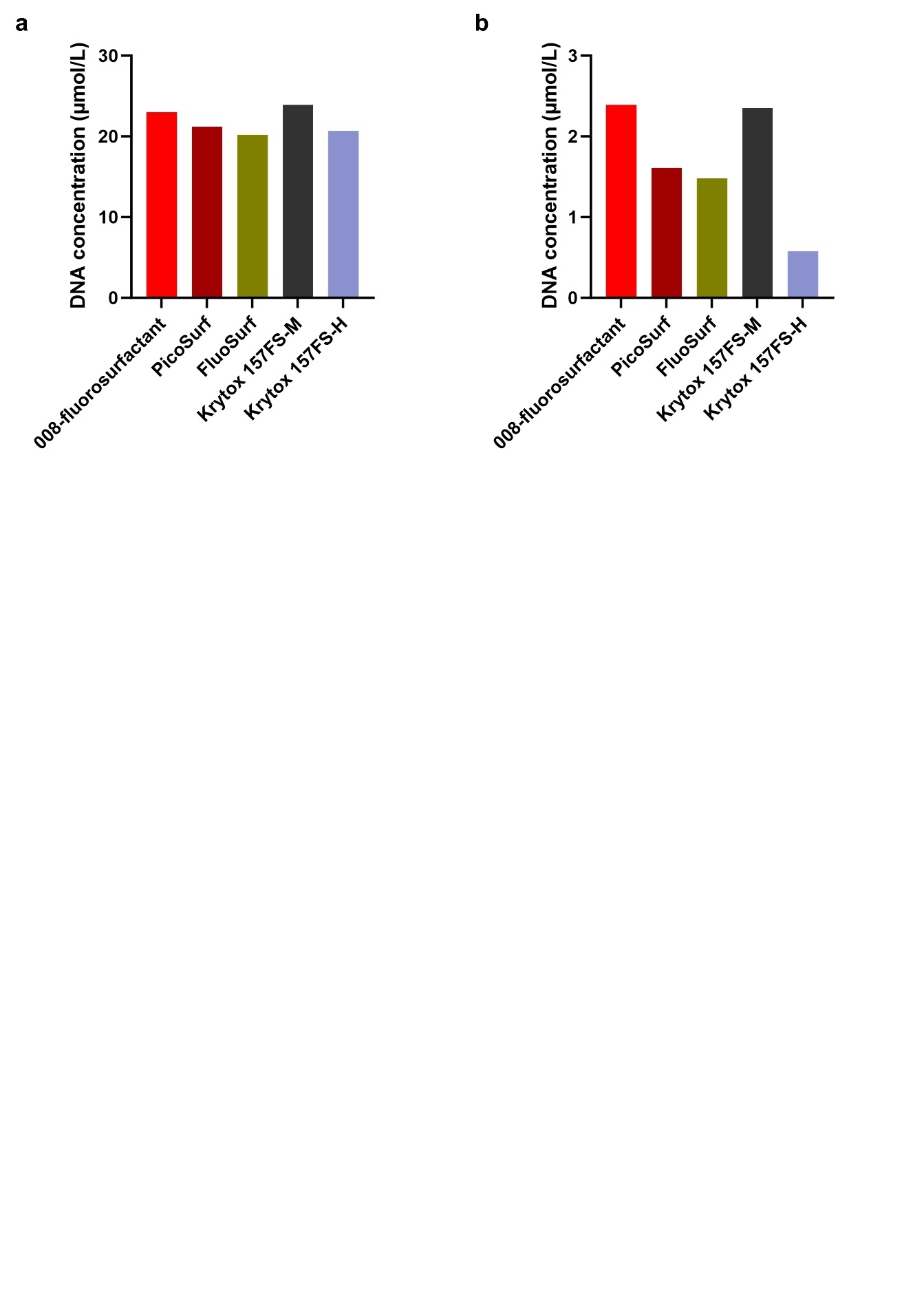
**

Figure S1. Oleophilic surfactant screening. DEs containing FAM-DNA were prepared using five different oleophilic surfactants, and the encapsulation capability (a) and release characteristics (b) of the resulting DEs were evaluated. a, The initial fluorescence intensity of the DEs was compared to a standard curve to quantify the amount of encapsulated DNA. b, The emulsions were incubated at 90°C for 10 min, and the amount of released FAM-DNA was measured and quantified. These findings revealed that the encapsulation and release at 90°C was not significantly different among DEs prepared with these different oleophilic surfactants in our setting, although 008-fluosurfactant and Krytox 157FS-M are slightly better than others.


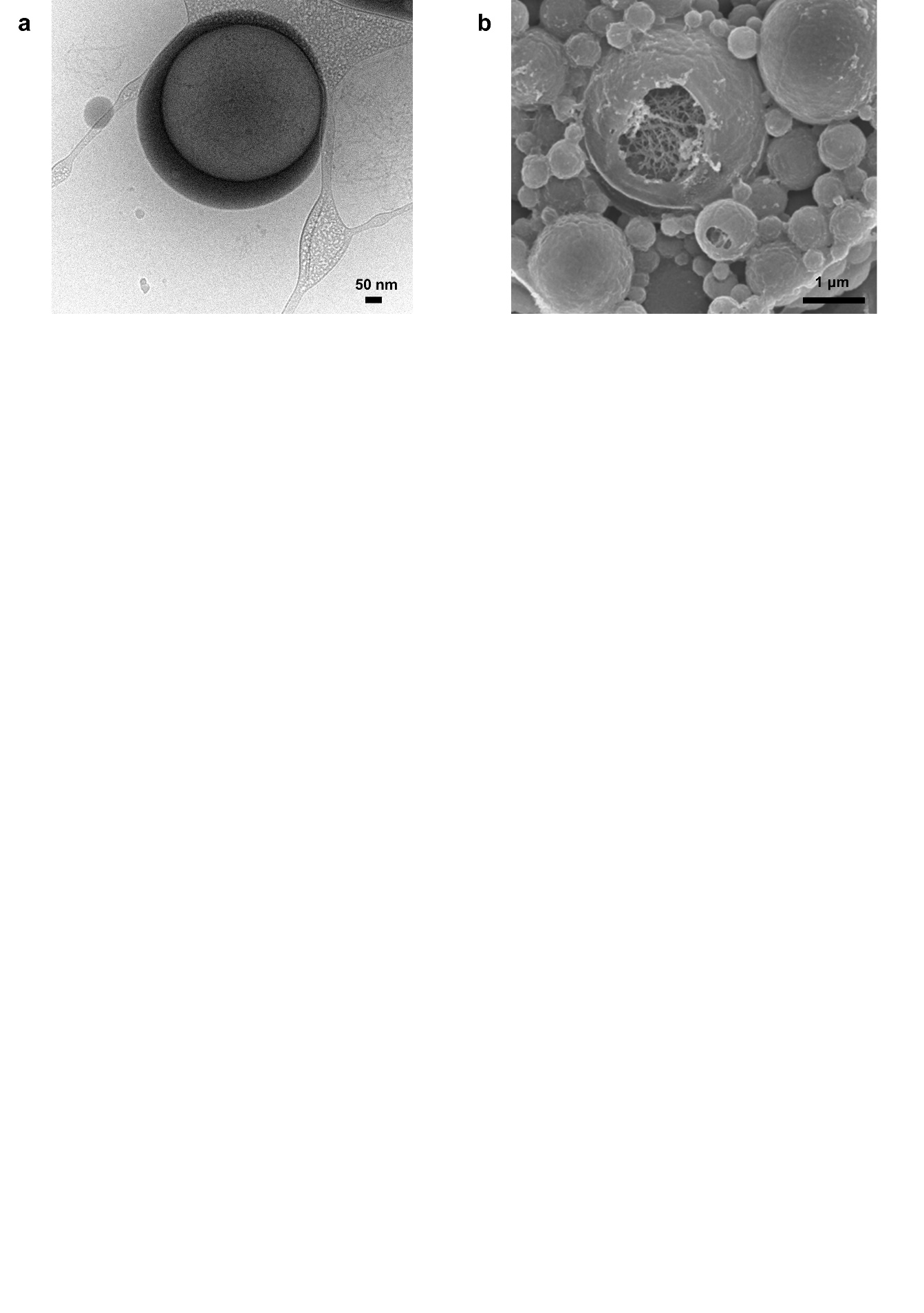


Figure S2. Cryo-TEM and cryo-SEM analyses of DEs. a, A TEM image of a water-in-oil-in-water DE. b, An SEM image of the DEs. An LMPA gel network formed in an inner water phase was shown.


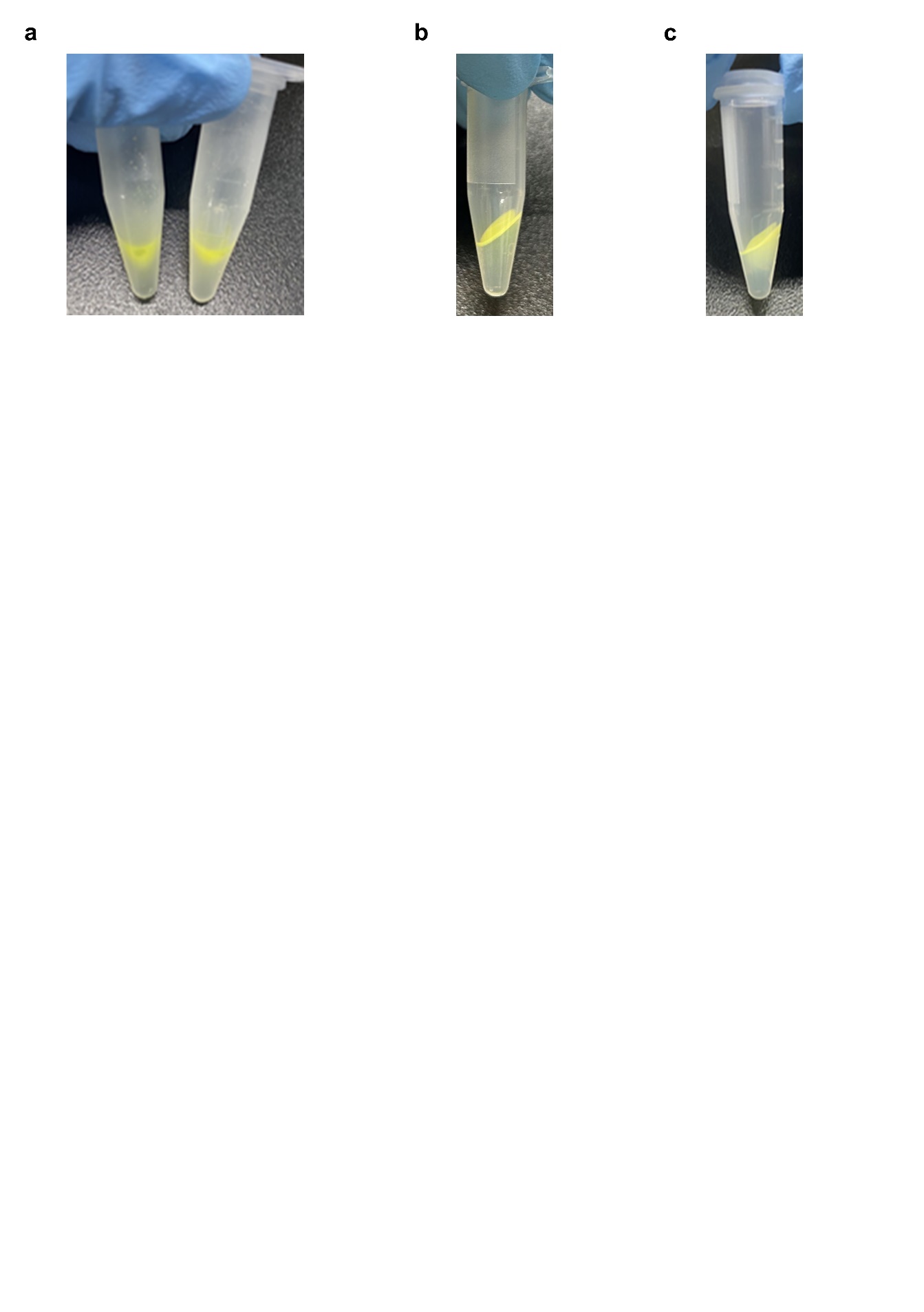


Figure S3. Preparation of W1/O single emulsions with varying amounts of oleophilic surfactant. a, An image showing the emulsification results using oleophilic surfactant at 0.05% (1 CMC). Tip sonication of water and oil failed to produce a stable emulsion due to insufficient surfactant amount. b, Image of W1/O single emulsions separated from the continuous oil phase. Emulsions formed with oleophilic surfactant concentrations ranging from 0.1% to 0.4% showed clear separation. (top yellow layer) c, In contrast, emulsions formed with the surfactant concentrations equal to orgreater than 0.6% showed no distinct separation from the oil phase.


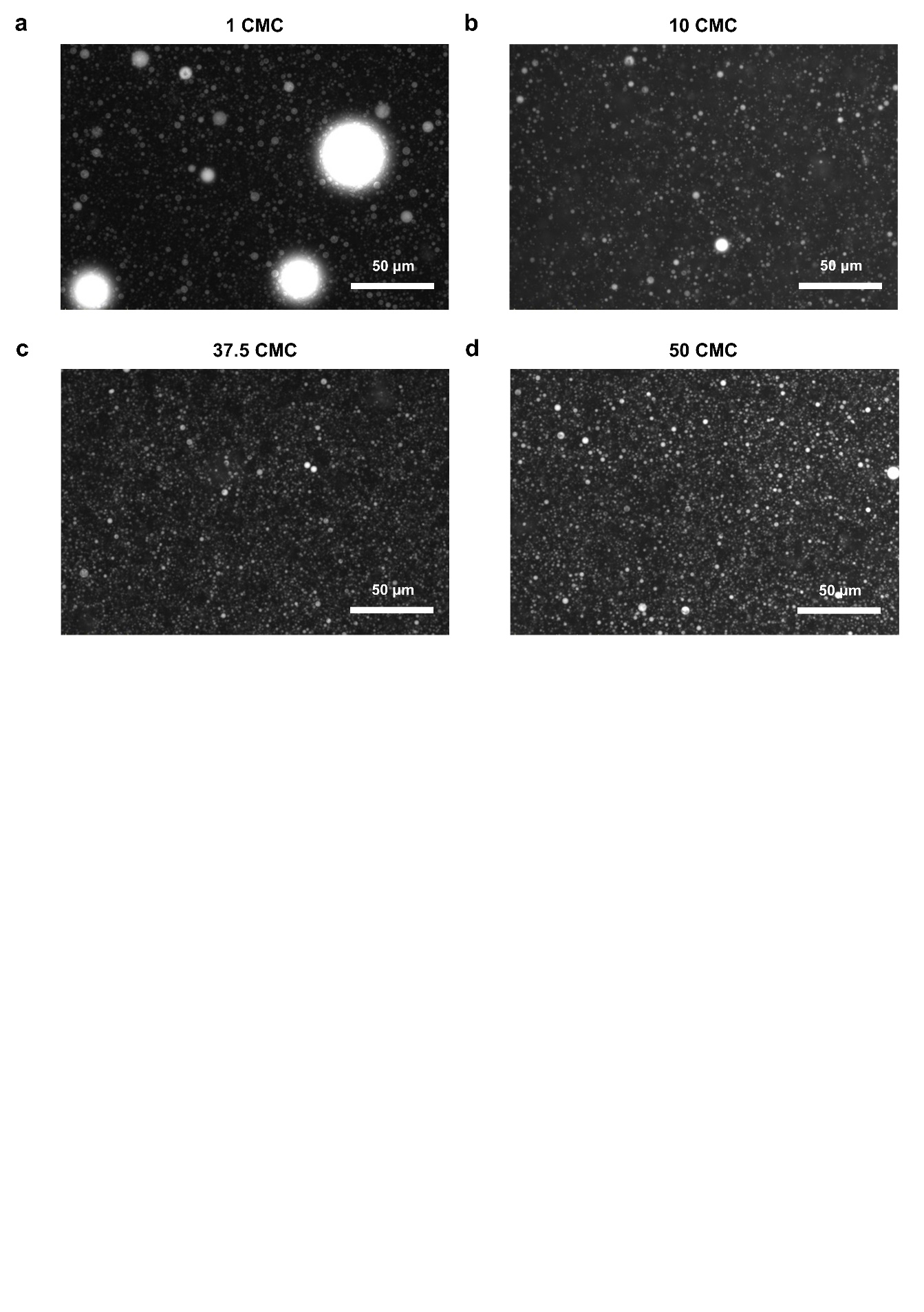


Figure S4. Images of W1/O/W2 DEs prepared with varying amounts of hydrophilic surfactant. a, Unstable formation of DEs due to insufficient amount of hydrophilic surfactant (1CMC) resulted in the production of large DEs. b – d, Hydrophilic surfactants at concentrations between 10 and 50 CMC led to the stable production of emulsions with a constant size distribution.


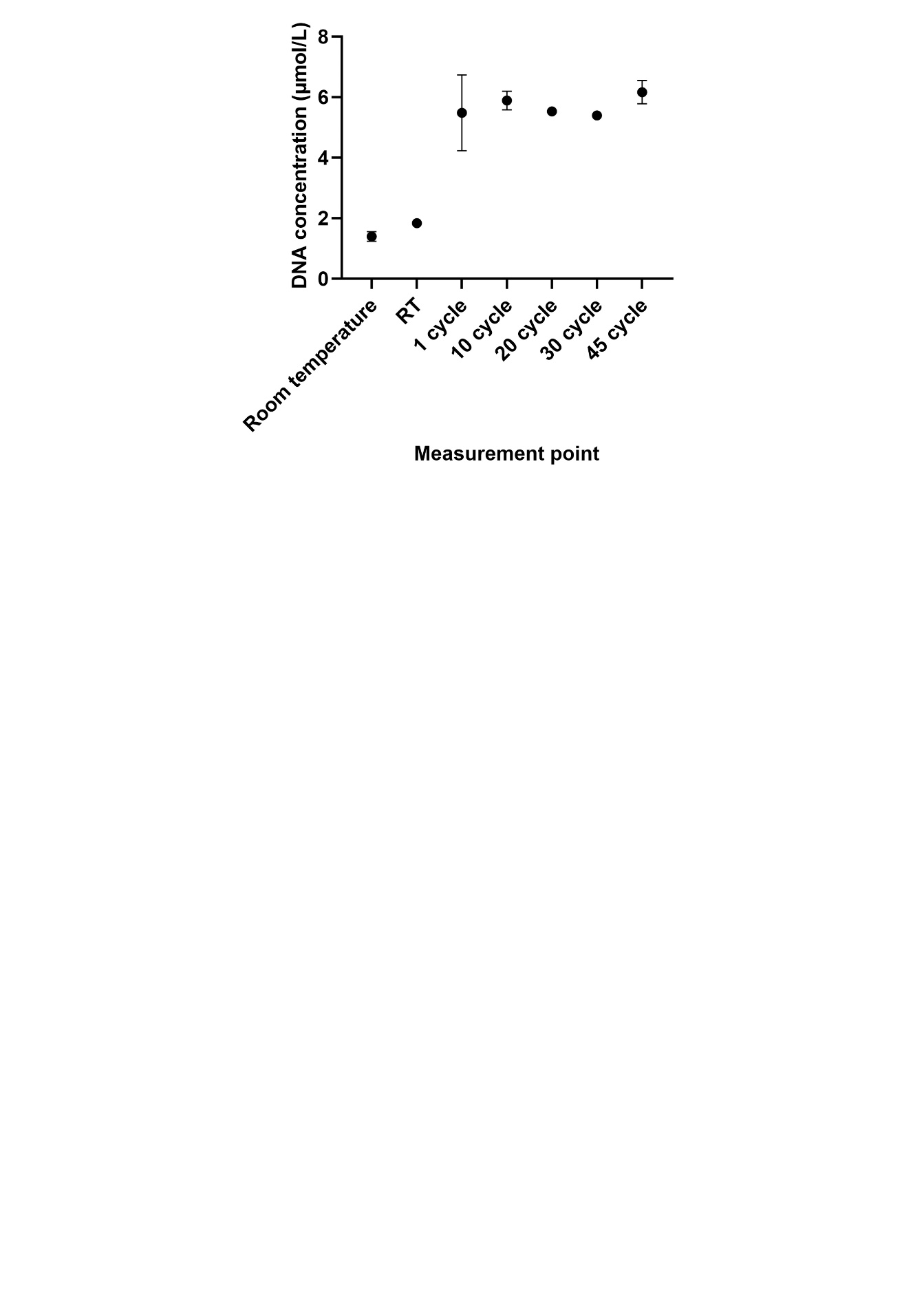


Figure S5. Release test of FAM-DNA on DEs during thermal cycling. We performed the release test under thermal cycling for RT-PCR. As a result, the majority of releasable FAM-DNAs were released during the first high-temperature cycle of PCR (1 cycle). In contrast, the amount released during the RT step was similar to that during room temperature storage, likely due to the measurement method to use centrifugation.


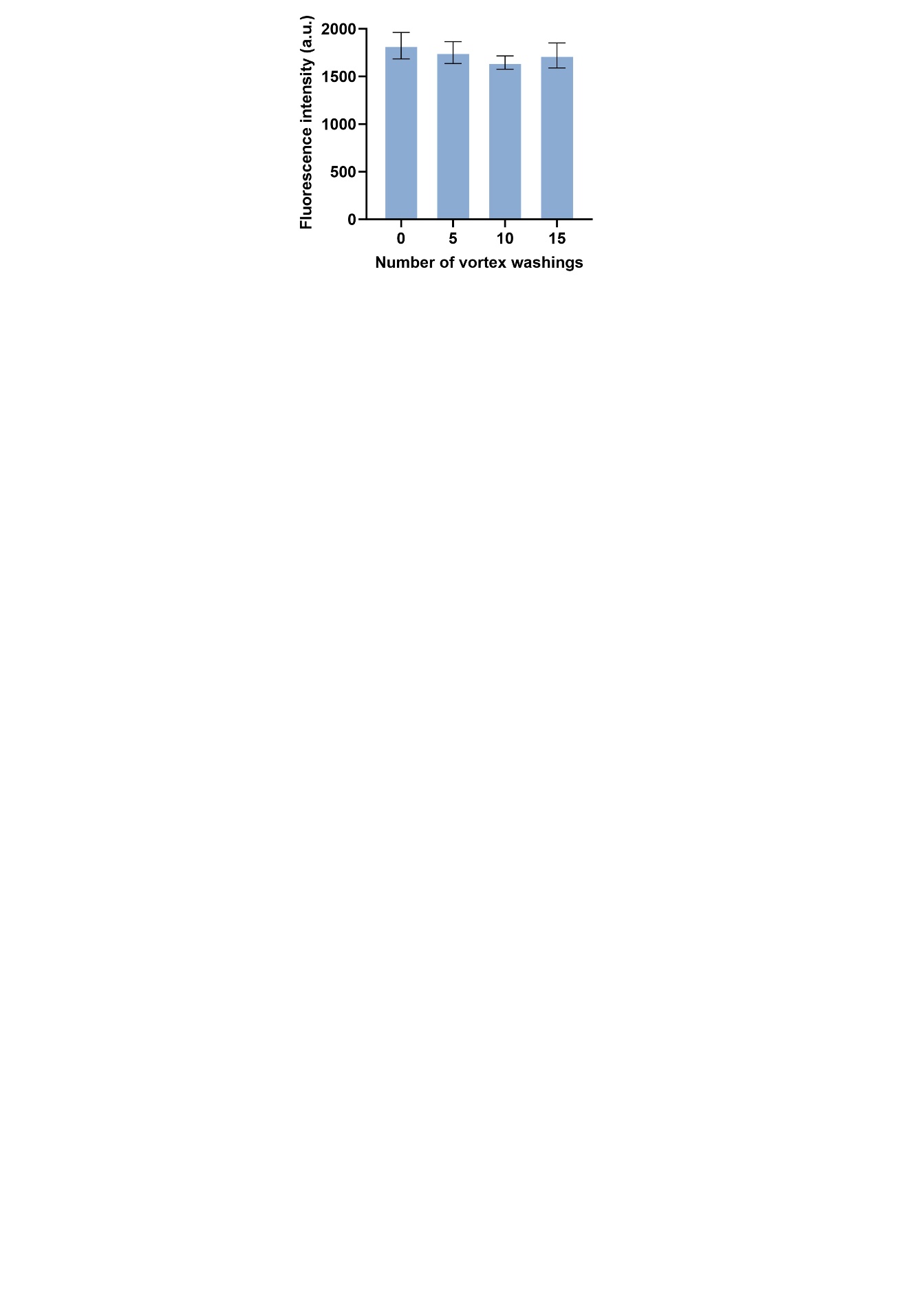


Figure S6. Stability of FAM-modified DNA-encapsulated DEs in hydrogel. The retention of DNA within DEs trapped in the hydrogel network remained similar, even after a stringent washing process (n=20).


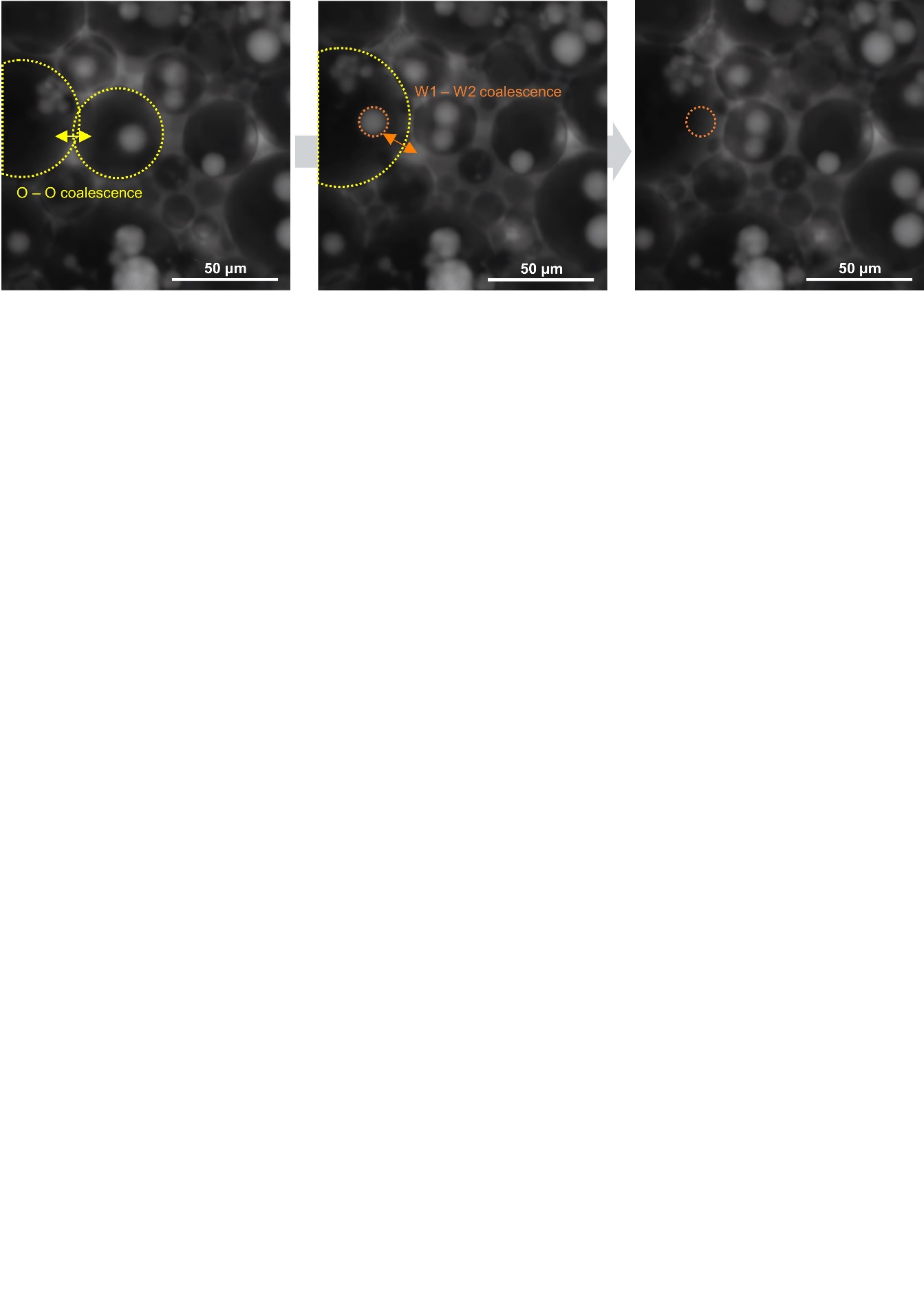


Figure S7. Thermal destabilization of micro-sized DEs. Relatively large DEs were fabricated through mild vortexing instead of ultrasonication, resulting in DEs with tens to hundreds of microns in diameter. DEs arranged in monolayer were heated on the hotplate from room temperature to 100℃ with the ramping rate of 0.2℃/sec as recording the fluorescence in real time. Real-time monitoring confirmed the coalescence of oil droplets (yellow) and the coalescence between the internal and external water phases (orange). Interestingly, with whole temperature range tested here, no coalescence between W1 droplets was observed, which might be related to the existence of mechanically supportive gel withstanding the necessary transformation process for merging.


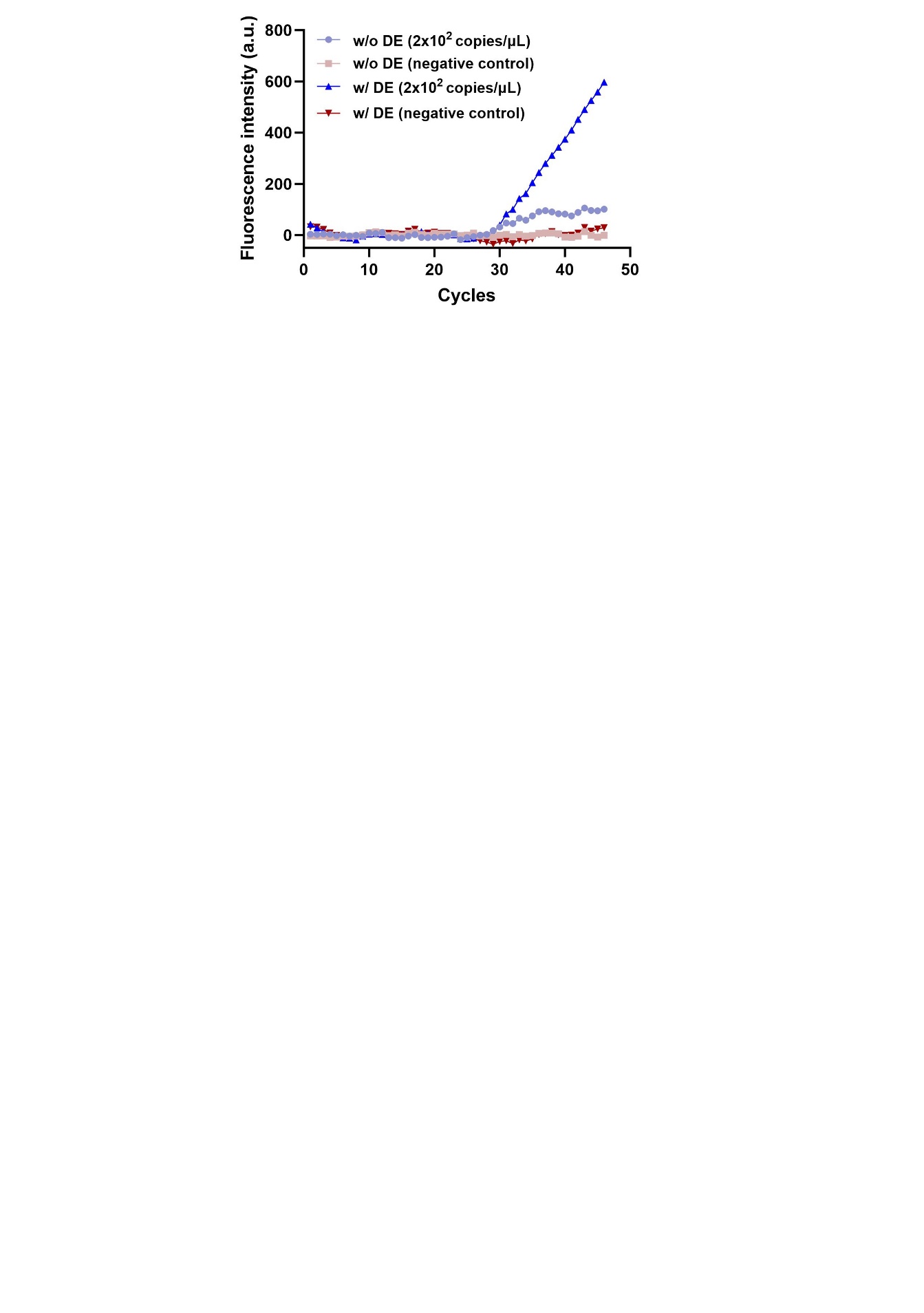


Figure S8. Increased sensitivity due to suppression of non-specific amplifications in the reactions containing DEs. The use of DEs in RT-qPCR enabled the detection of a less-abundant target (2x10^2^ copies/μL) whereas conventional RT-qPCR failed to elicit a fluorescence signal from the identical target. This may be attributed to the suppression of non-specific amplifications, which leads to be the erroneous consumption of the reagents.


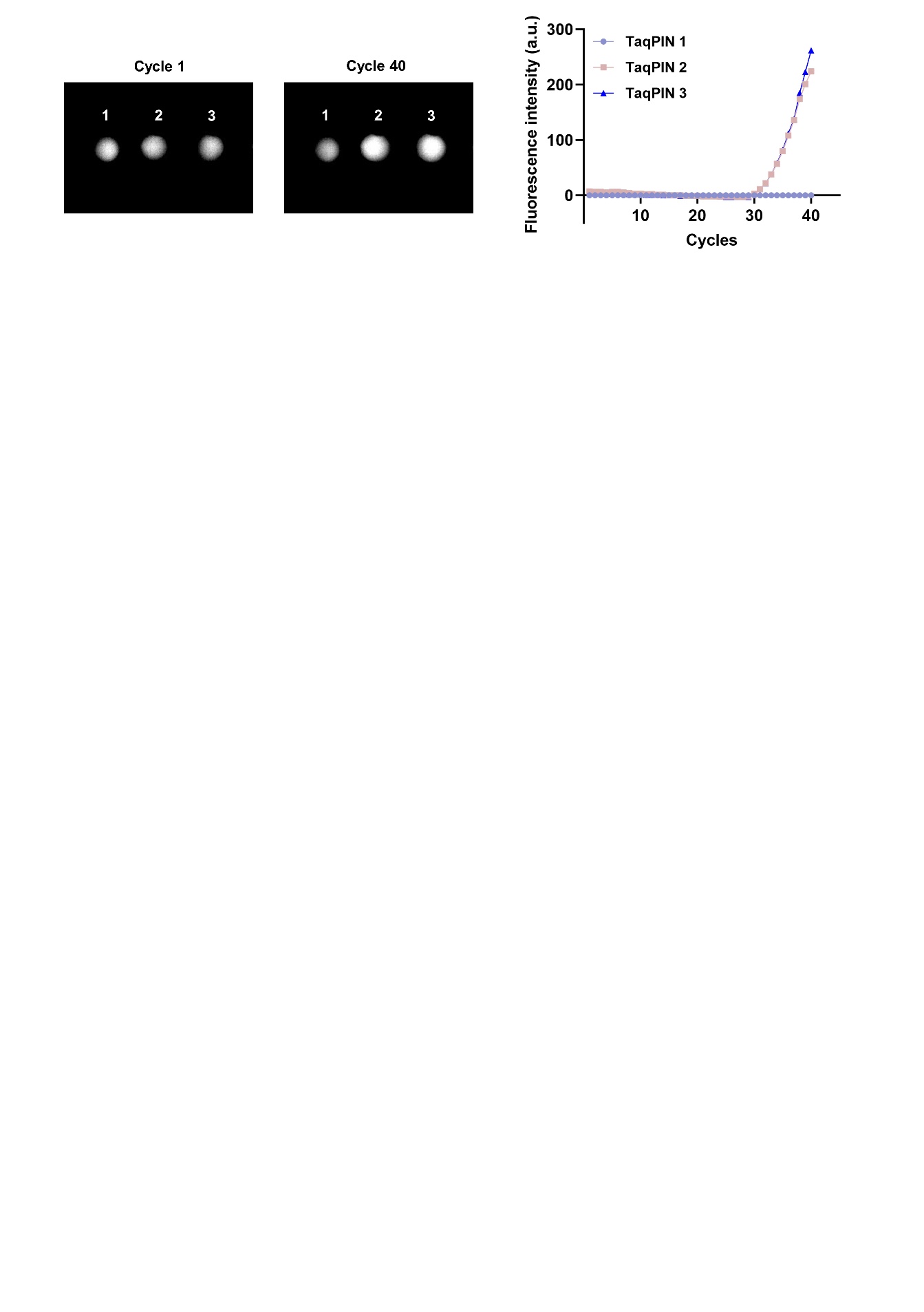


**Figure S9. Detection of a low-concentration sample (6.2 copies/μL) using the TaqPIN array.** Only two of three TaqPINs (TaqPIN 2 and TaqPIN 3) on one microfluidic chip showed amplification signals, as not all TaqPINs contained the target nucleic acid. Images of the TaqPIN array taken after the first and the final PCR cycle are shown (left). The fluorescence intensity of each TaqPIN was recorded at every cycle and plotted against cycle number (right).


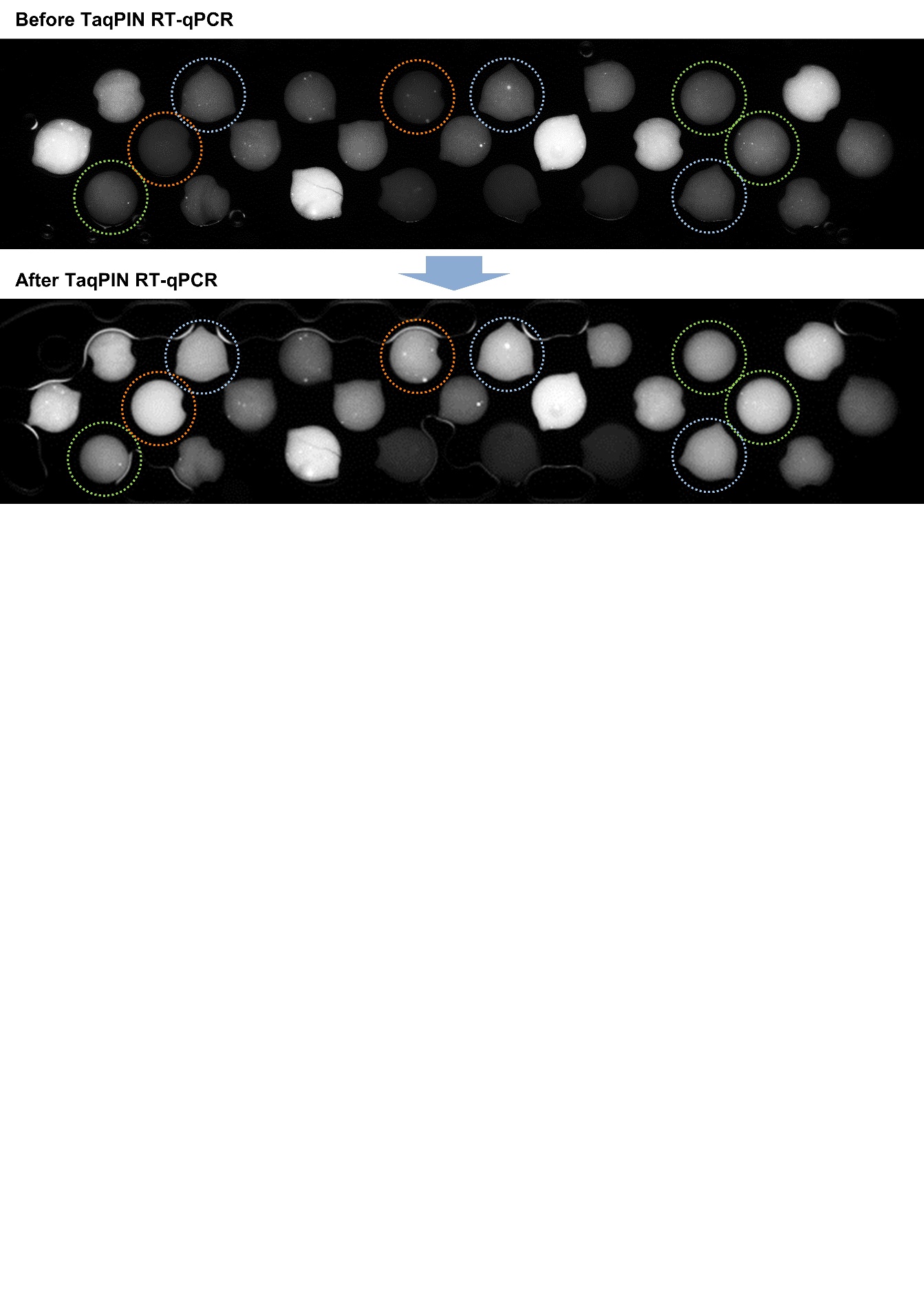


Figure S10. Multiplexed RT-qPCR detecting Delta variant sample with an array of 25 TaqPINs. A total of 25 TaqPINs were arrayed in a single chip. Two TaqPINs each of S 69-70del dropout and S 157-158del dropout were loaded, along with three microparticles each of the other seven types of TaqPINs. TaqPINs showing a brightening after RT-qPCR were marked with colored circles (green, orange, and blue for TaqPINs detecting N gene, S 69-70del dropout, and S 157-158del, respectively). This representation is sufficient to identify the Delta variant.


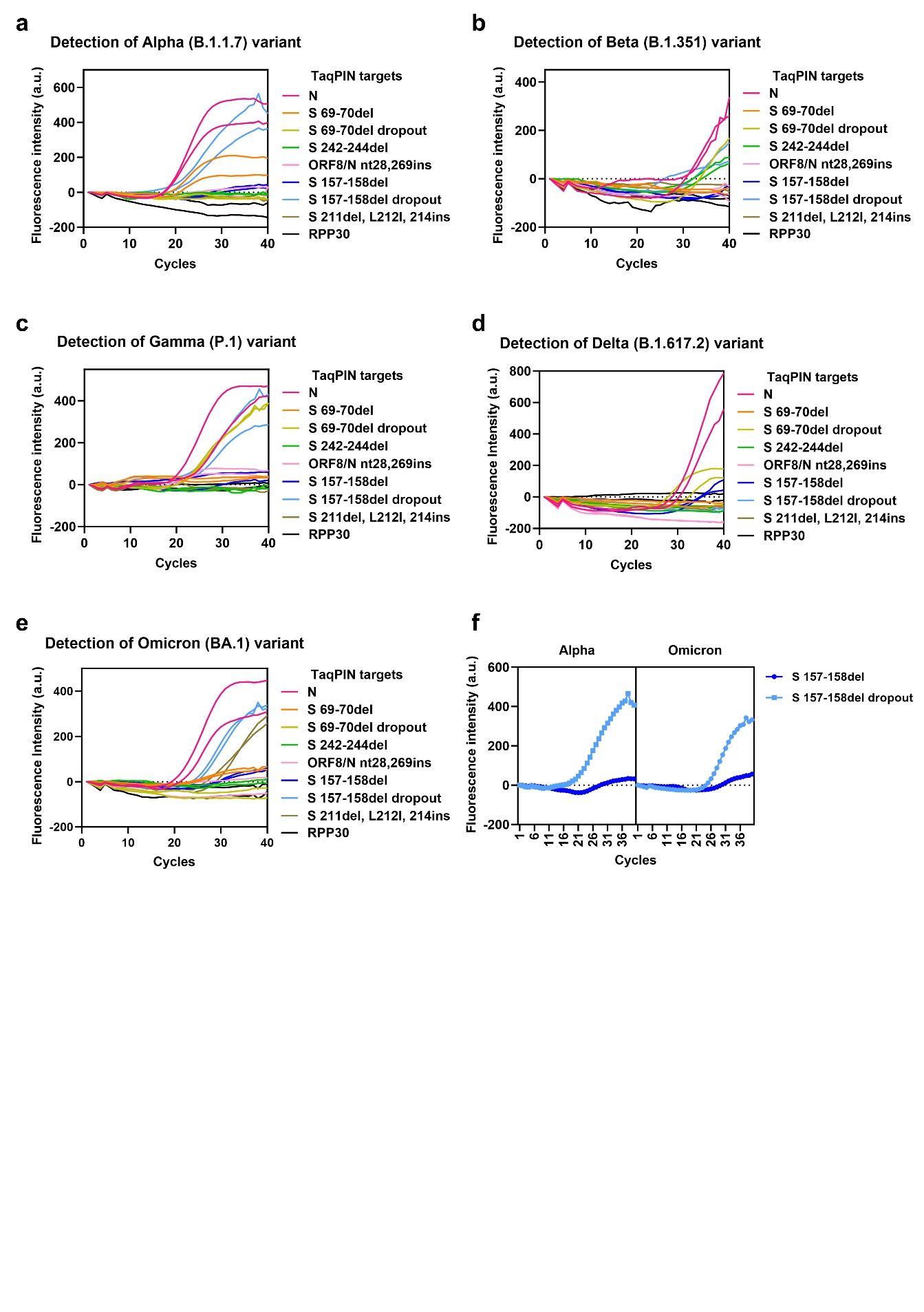


Figure S11. Discrimination of SARS-CoV-2 variants using TaqPIN RT-qPCR. The RT-qPCR results detecting the Alpha (a), Beta (b), Gamma (c), Delta (d), and Omicron (e) variants are shown. TaqPINs yielding a fluorescent signal for each variant detection were marked in green. f, Fluorescence signals of the S 156-157del- and S 156-157del dropout-targeting TaqPINs tested with alpha and omicron variants. A weak fluorescence signal was detected from the S 156-157del-targeting TaqPIN, but the S 156-157del dropout-targeting TaqPIN showed a much stronger signal, confirming that the targets were not Delta variant.


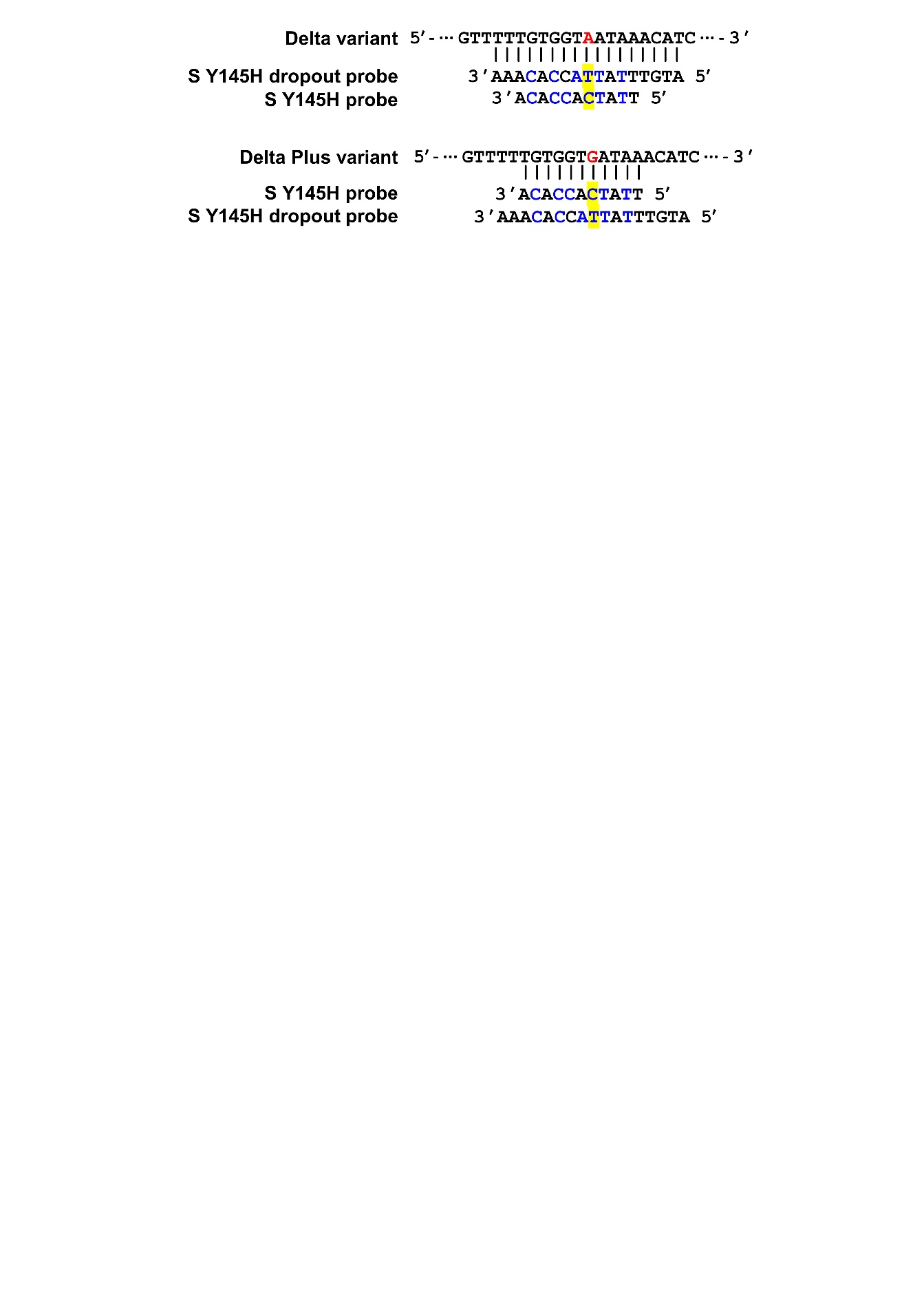


Figure S12. LNA probe design for precise discrimination of single nucleotide variation in Delta Plus variant. Single nucleotide variations in cDNA sequences of two strains are highlighted in red. In the probe sequences, LNA bases are indicated in blue and DNA bases are indicated in black. The bases of the probes used for discriminating target mutations are highlighted in yellow.


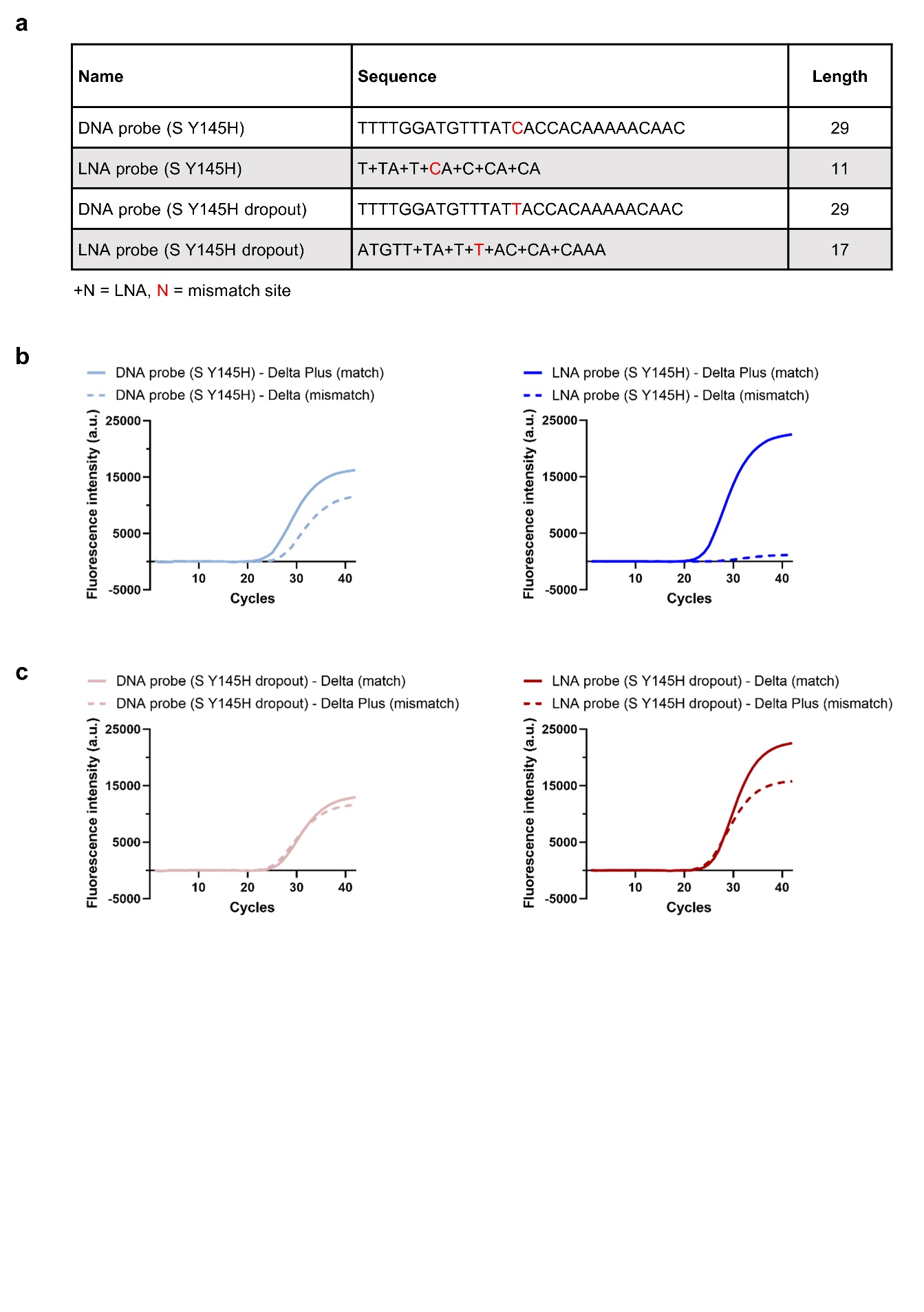


**Figure S13.** **Discrimination of a single nucleotide variation using LNA probes.** a. Sequence information of DNA probes and LNA probes for detecting S Y145H mutation and S Y145H dropout. The target mutation is marked in red. b-c, The qPCR results showing the single nucleotide variation discrimination of a DNA probe and an LNA probe designed to detect the S Y145H mutation (b) and S Y145H dropout (c).

**Supporting tables**

Table S1. DNA encapsulation and release efficiency of DEs as a function of the amount of oleophilic surfactant.

| Oleophilic  surfactant conc. | | Fluorescence intensity^a^ | | FAM-DNA  conc.  (μmol/L)^b^ | Diameter of DEs^c^ | | DNA release efficiency (%)^d^ | |
| --- | --- | --- | --- | --- | --- | --- | --- | --- |
| % | CMC | Mean (a.u.) | SD (a.u.) |  | mean (nm) | SD (nm) | at 55 ℃ | at 95 ℃ |
| 0.05 | 1 | 3950 | 169 | 3.55 | 916.9 | 154.8 | 30.7 | 80.9 |
| 0.1 | 2 | 6753 | 136 | 6.07 | 847.1 | 349.1 | 19.1 | 57.7 |
| 0.2 | 4 | 8219 | 711 | 8.00 | 914.3 | 323.9 | 20.3 | 35.5 |
| 0.3 | 6 | 10712 | 433 | 10.49 | 938.7 | 169.9 | 9.3 | 15.2 |
| 0.4 | 8 | 16366 | 7 | 14.72 | 920.3 | 84.9 | 4.9 | 9.6 |
| 0.6 | 12 | 11007 | 923 | 9.99 | 960.0 | 51.4 | 7.9 | 10.4 |
| 0.8 | 16 | 13622 | 124 | 12.35 | 892.3 | 100.0 | 5.0 | 9.6 |
| 1 | 20 | 12541 | 601 | 12.32 | 854.4 | 147.3 | 2.3 | 4.6 |
| 2 | 40 | 12394 | 181 | 11.15 | 1022.0 | 106.4 | 2.6 | 4.6 |

Conc., concentration; CMC, critical micelle concentration; FAM-DNA, fluorescein-labeled DNA; DEs, double emulsions; SD, standard deviation

^a^Fluorescence intensities (a.u.) were measured using fluorescent spectrophotometry.

^b^Concentrations of FAM-DNA encapsulated in DEs were estimated by fitting the measured fluorescence intensity to a standard curve.

^c^Diameters of DEs were measured by DLS.

^d^The fraction of fluorescence decrease in DEs after exposure to the indicated temperature, relative to the encapsulated FAM-DNA concentration, was expressed.

Table S2. DNA encapsulation and release efficiency of DEs as a function of the amount of hydrophilic surfactant.

| Hydrophilic  surfactant conc. | | Fluorescence intensity | | FAM-DNA  conc.  (μmol/L) | Diameter of DEs | | DNA release efficiency (%) | |
| --- | --- | --- | --- | --- | --- | --- | --- | --- |
| mmol/L | CMC | mean | SD |  | mean (nm) | SD (nm) | at 55 ℃ | at 95 ℃ |
| 0.06 | 1 | 5068 | 879.39 | 2.37 | 1005 | 28 | 28.3 | 53.6 |
| 0.6 | 10 | 22943 | 583.95 | 12.13 | 1087 | 104 | 4.0 | 7.6 |
| 2.2 | 37.5 | 19686 | 75.95 | 10.30 | 954 | 75 | 4.5 | 9.3 |
| 3 | 50 | 20012 | 556.57 | 10.49 | 799 | 62 | 4.5 | 8.8 |

Table S3. Sequences of primers and probes used for the detection of SARS-CoV-2 variants.

| **Strain** | **Targets** | **Primers** | **Sequence** | **Length** | **Reference** |  |
| --- | --- | --- | --- | --- | --- | --- |
| **All SARS-CoV-2** | **N gene** | **F** | GGGAGCCTTGAATACACCAAAAG | **23** | ^[15]^ |  |
|  |  | **R** | TGTAGCACGATTGCAGCATTG | **21** |  |  |
|  |  | **P** | TCACATTGGCACCCGCAATCCTGC | **24** |  |  |
| **Alpha (B.1.1.7)** | **S 69-70del** | **F** | TCAACTCAGGACTTGTTCTTACCT | **24** | ^[15]^ |  |
|  |  | **R** | TGGTAGGACAGGGTTATCAAAC | **22** |  |  |
|  |  | **P** | TCCATGCTATCTCTGGGACC | **20** |  |  |
|  |  | **Dropout P^a^** | TTCCATGCTATACATGTCTCTGGGA | **25** |  |  |
| **Beta (B.1.351)** | **S 242-244del** | **F** | AGATTTGCCAATAGGTATTAACATC | **25** | ^[15]^ |  |
|  |  | **R** | CTGAAGAAGAATCACCAGGAGTC | **23** |  |  |
|  |  | **P** | CTAGGTTTCAAACTTTACATAGAAGTT | **27** |  |  |
| **Gamma (P.1)** | **ORF8/N nt28,269ins** | **F** | CATGACGTTCGTGTTGTTTTAG | **22** | ^[16]^ |  |
|  |  | **R** | CATTTCGCTGATTTTGGGGTCC | **22** |  |  |
|  |  | **P** | TTTCATCTAAACGAACAAACAAACTAAAAT | **30** |  |  |
| **Delta (B.1.617.2)** | **S 157-158del** | **F** | GTTTATTACCACAAAAACAACAAAAG | **26** | ^[16]^ |  |
|  |  | **R** | GGCTGAGAGACATATTCAAAAGTG | **24** |  |  |
|  |  | **P** | TGGATGGAAAGTGGAGTTTATTCTAGT | **27** |  |  |
|  |  | **Dropout P** | TGGAAAGTGAGTTCAGAGTTTATT | **24** |  |  |
| **Omicron (BA.1)** | **S 211del, L212I, 214ins** | **F** | AATCTTAGGGAATTTGTGTTTAAGA | **25** |  |  |
|  |  | **R** | TCTAAAGCCGAAAAACCCT | **19** |  |  |
|  |  | **P** | TTATAGTGCGTGAGCCAGAAGAT | **23** |  |  |
| **Delta Plus**  **(AY.4.2)** | **S Y145H** | **F** | GTAATGATCCATTTTTGGATGTTTAT | **26** |  |  |
|  |  | **R** | AGAGACATATTCAAAAGTGCAAT | **23** |  |  |
|  |  | **P^b^** | T+TA+T+CA+C+CA+CA | **11** |  |  |
|  |  | **Dropout P** | ATGTT+TA+T+T+AC+CA+CA AA | **17** |  |  |
|  | **RPP30 gene** | **F** | AGATTTGGACCTGCGAGCG | **19** |  |  |
|  |  | **R** | GAGCGGCTGTCTCCACAAGT | **20** |  |  |
|  |  | **P** | TTCTGACCTGAAGGCTCTGCGCG | **23** |  |  |
| ^a^Sequence of the S 69-70del probe is modified in this study; ^b^+N = LNA | | | | | |  |

Table S4. Distribution of Ct values of positive samples used for clinical tests.

| **Ct range** | **Number of samples** | **Portion (%)** |
| --- | --- | --- |
| Ct<20 | 3 | 4.5 |
| 20≤Ct<25 | 24 | 36.4 |
| 25≤Ct<30 | 24 | 36.4 |
| 30≤Ct | 15 | 22.7 |
| Total | 66 | 100.0 |

Table S5. Comparative analysis of the detection methods used for SARS-CoV-2 and its variants.

|  | **mCARMEN [3]** | **SHINEv.2 [4]** | **MARVE [5]** | **This study** |
| --- | --- | --- | --- | --- |
| **Chip** | Fluidigm microfluidics | Paper strip | Paper strip | Hydrogel array |
| **Assay principle** | RT-qPCR + Cas12 or Cas13 | RT-RPA + Cas13 | Strand displacement | RT-qPCR |
| **Reaction time** | 110 min | 90 min | 20 min | **38 min** |
| **Reaction complexity** | Two-step | One-step | One-step | **One-step** |
| **Target strains per a reaction** | 6 | 1 | 5 | **6** |
| **Analytical sensitivity** | 100 copies/μL | 200 copies/μL | 400 copies/μL | **20 copies/μL** |
| **Clinical sensitivity** | 100% | 90.5% | 100% | 100% |
| **Clinical specificity** | 100% | 100% | 100% | 100% |
| **Maximum gene targets in a reaction** | 96 (manual injection) | 2 | 13 | **25** |
|  | | |  |  |

**References**

[1] Yaniv, K. et al. Direct RT-qPCR assay for SARS-CoV-2 variants of concern (Alpha, B.1.1.7 and Beta, B.1.351) detection and quantification in wastewater. *Environ Res* **201**, 111653 (2021).

[2] Yaniv, K., Ozer, E., Lewis, Y. & Kushmaro, A. RT-qPCR assays for SARS-CoV-2 variants of concern in wastewater reveals compromised vaccination-induced immunity. *Water Res* **207**, 11 (2021).

[3] Welch, N. L. et al. Multiplexed CRISPR-based microfluidic platform for clinical testing of respiratory viruses and identification of SARS-CoV-2 variants. *Nat Med* **28,** 1083 (2022).

[4] Arizti-Sanz, J. et al. Simplified Cas13-based assays for the fast identification of SARS-CoV-2 and its variants. *Nat Biomed Eng* **6,** 932 (2022).

[5] Zhang T. et al. A paper-based assay for the colorimetric detection of SARS-CoV-2 variants at single-nuleotide resolution. *Nat Biomed Eng* **6,** 957 (2022).
